# Global mortality associated with seasonal influenza epidemics: New burden estimates and predictors from the GLaMOR Project

**DOI:** 10.7189/jogh.09.020421

**Published:** 2019-12

**Authors:** John Paget, Peter Spreeuwenberg, Vivek Charu, Robert J Taylor, A Danielle Iuliano, Joseph Bresee, Lone Simonsen, Cecile Viboud, for the Global Seasonal Influenza-associated Mortality Collaborator Network and GLaMOR Collaborating Teams

**Affiliations:** 1 Netherlands Institute for Health Services Research (NIVEL), Utrecht, the Netherlands; 2 Centers for Disease Control and Prevention, Atlanta, Georgia, USA; 3 Fogarty International Center, National Institutes of Health, Bethesda, Maryland, USA; 4 Stanford University, Stanford, California, USA; 5 Sage Analytica, Bethesda, Maryland, USA; 6 George Washington University, Washington, D.C., USA; 7 Roskilde University, Roskilde, Denmark; * Members of the Global Seasonal Influenza-associated Mortality Collaborator Network and the GLaMOR Collaborating Teams are listed in the Acknowledgements

## Abstract

**Background:**

Until recently, the World Health Organization (WHO) estimated the annual mortality burden of influenza to be 250 000 to 500 000 all-cause deaths globally; however, a 2017 study indicated a substantially higher mortality burden, at 290 000-650 000 influenza-associated deaths from respiratory causes alone, and a 2019 study estimated 99 000-200 000 deaths from lower respiratory tract infections directly caused by influenza. Here we revisit global and regional estimates of influenza mortality burden and explore mortality trends over time and geography.

**Methods:**

We compiled influenza-associated excess respiratory mortality estimates for 31 countries representing 5 WHO regions during 2002-2011. From these we extrapolated the influenza burden for all 193 countries of the world using a multiple imputation approach. We then used mixed linear regression models to identify factors associated with high seasonal influenza mortality burden, including influenza types and subtypes, health care and socio-demographic development indicators, and baseline mortality levels.

**Results:**

We estimated an average of 389 000 (uncertainty range 294 000-518** **000) respiratory deaths were associated with influenza globally each year during the study period, corresponding to ~ 2% of all annual respiratory deaths. Of these, 67% were among people 65 years and older. Global burden estimates were robust to the choice of countries included in the extrapolation model. For people <65 years, higher baseline respiratory mortality, lower level of access to health care and seasons dominated by the A(H1N1)pdm09 subtype were associated with higher influenza-associated mortality, while lower level of socio-demographic development and A(H3N2) dominance was associated with higher influenza mortality in adults ≥65 years.

**Conclusions:**

Our global estimate of influenza-associated excess respiratory mortality is consistent with the 2017 estimate, despite a different modelling strategy, and the lower 2019 estimate which only captured deaths directly caused by influenza. Our finding that baseline respiratory mortality and access to health care are associated with influenza-related mortality in persons <65 years suggests that health care improvements in low and middle-income countries might substantially reduce seasonal influenza mortality. Our estimates add to the body of evidence on the variation in influenza burden over time and geography, and begin to address the relationship between influenza-associated mortality, health and development.

Until late 2017, WHO estimated that seasonal influenza was associated with a total of 250 000 to 500 000 deaths from all causes annually [1]. Recently, however, three different groups have provided estimates of influenza's annual mortality burden using different methods. The first to be published, from the US Centers for Disease Control and Prevention (CDC) and coordinated by WHO, estimated that influenza is associated with 290 000 to 650 000 deaths from respiratory causes alone [2]. WHO adopted this range in late 2017 as its official assessment [2]. In early 2019, a publication from the Global Burden of Disease Study (GBD) estimated a range of 99 000 to 200 000 annual deaths from lower respiratory tract infections directly attributable to influenza [3]. Although the burden of influenza is known to fluctuate greatly between years, neither of these estimates were broken down by year or circulating strains.

We report here a third estimate, from the Global Influenza Mortality project (GLaMOR), of 294 000 to 518 000 influenza-associated respiratory deaths annually. Our study, like the CDC study, was coordinated by WHO but our extrapolation model did not rely on the same assumptions, particularly that influenza-associated mortality scales with respiratory death rates. We used national vital statistics data to assess influenza-associated respiratory mortality in 31 countries, then used a multiple imputation approach to extrapolate those estimates to the WHO-regional and global levels. Our approach allowed us to make individual annual estimates for the period 2002-2011 (excluding the 2009 pandemic year) while stratifying by age (0-64 and 65+ years). Moreover, we identified factors associated with high seasonal influenza mortality burden, including circulation of influenza A subtypes, demography, health and development indicators.

## METHODS

We used a two-stage modelling approach to estimate the global respiratory mortality burden of influenza, which we have described in detail in prior work focused on the 2009 influenza pandemic [4] and **Online Supplementary Document**. In Stage 1, we generate annual age-specific estimates of influenza associated deaths from respiratory causes in a subset of countries with available weekly or monthly national statistics. In Stage 2, we extrapolate these estimates to the world population, and explore virologic, geographic, and socio-demographic predictors of mortality.

### Stage 1

In Stage 1, we used multi-year age-specific excess respiratory mortality rates (<65 years and ≥65 years) from 30 of the 33 countries included in a previous CDC study [2]. Annual age- and country-specific estimates were obtained from time series models applied to vital statistics data on respiratory deaths and influenza surveillance indicators, using various model forms and assumptions (**Table 1**). We estimated influenza-associated mortality for two additional countries (Sweden and Poland) that were not in the CDC-led study [2] and updated estimates to represent all of Brazil; for these 3 countries we ran weekly time series models including flexible seasonal baselines and influenza viral proxies (**Table 1**). We used 31 countries in our final Stage 1 sample, which represented 37% of the world population and included countries from five of the six WHO regions. The sample was skewed towards Europe, Western Pacific and the Americas. We conducted sensitivity analyses with a larger set of 33 countries, with the addition of India and Kenya, for which influenza-associated mortality estimates were derived from a population sample rather than national vital statistics data (see **Online Supplementary Document** for more details). To calculate annual excess mortality rates, we used a definition of “respiratory year” tailored to influenza circulation, representing July 1 to June 30 for countries in the Northern hemisphere, and the calendar year (January 1 to December 31) for countries in the Southern Hemisphere or countries with tropical climate.

**Table 1:** Participating countries for which estimated influenza-associated respiratory mortality (Stage 1) were used in the global projection (Stage 2)*

WHO region (number of countries)	Country	Data years	Number of years/seasons	Modelling method	Percent world population (2011)^†^
**Europe (15)**	Austria	1999/2000-2009/2010	11	Poisson regression	0.1%
	Czech Republic	1999/2000-2010/2011	12	Negative binomial	0.2%
	Denmark	2002/2003-2013/2014	12	Negative binomial	0.1%
	Germany	2002/2003-2014/2015	13	Linear generalised additive	1.2%
	Israel	2004/2004-2013/2014	10	Negative binomial	0.1%
	Netherlands	1999/2000-2010-2011	12	Generalised linear	0.2%
	Norway	1999/2000-2014/2015	16	Poisson regression	0.1%
	Portugal	1999/2000-2013/2014	14	Linear regression with Serfling	0.2%
	Poland	2002/2003-2014-2015	13	Negative binomial	0.5%
	Romania	2000/2001-2013/2014	14	Negative binomial	0.3%
	Serbia	1999/2000-2010/2011	12	Linear regression with Serfling	0.1%
	Spain	2000/2001-2012/2013	13	Negative binomial	0.7%
	Sweden	2003/2004-2014/2015	12	Negative binomial	0.1%
	Switzerland	1999/2000-2013/2014	15	Negative binomial	0.1%
	United Kingdom^‡^	2006/2007-2011/2012	6	Poisson regression	0.9%
**Americas (8)**	Argentina	2001-2009	9	Linear regression with Serfling	0.6%
	Brazil	2004-2015	12	Negative binomial	2.8%
	Canada	1999/2000-2007/2008	9	Poisson regression	0.5%
	Chile	2002-2009	8	Linear regression with Serfling	0.2%
	Mexico	2002/2003-2009/2010	8	Linear regression with Serfling	1.6%
	Paraguay	2002-2009	8	Linear regression with Serfling	0.1%
	Uruguay	2004-2009	4	Linear regression with Serfling	0.0%
	USA	1981/1982-2014/15	34	Negative binomial	4.5%
**Western Pacific (6)**	Australia	2003-2009	7	Linear generalised additive with splines	0.3%
	Hong Kong	1999-2015	17	Generalised linear	0.1%
	China	2004/2005-2009/2010	6	Negative binomial	19.3%
	New Zealand	2002-2013	12	Negative binomial	0.1%
	Singapore	2004-2011	7	Negative binomial with splines	0.1%
	Rep. South Korea	2003-2011	10	Multiple linear regression	0.7%
**South-East Asia (1)**	Thailand	2006-2011	6	Negative binomial	1.0%
**Sub-Saharan Africa^§^ (1)**	South Africa	1999-2013	15	Poisson regression (10 years) & Generalized additive models with splines (5 years)	0.7%
**Sensitivity analysis:**					
**South-East Asia (1)**	India	2010-2013	4	Negative binomial	17.8%
**Sub-Saharan Africa (1)**	Kenya	2007-2013	7	Negative binomial	0.6%

*See Appendix S1 in **Online Supplementary Document** for more details about the models used in each country. Three (Brazil, Sweden and Poland) of the 33 Stage 1 estimates were generated in-house by the authors specifically for this project; the other estimates were generously contributed by the CDC co-authors and the country representatives listed in the GLaMOR group author list.

†World Population Data Set, 2011 (http://www.prb.org/pdf11/2011population-data-sheet_eng.pdf).

‡United Kingdom = England and Wales.

§Officially, the WHO region is “Africa” but we have defined it at “Sub-Saharan Africa” so that readers have a more precise definition of this region (North Africa is part of the WHO region “Eastern Mediterranean”).

### Stage 2

In Stage 2, we used a previously developed multiple imputation model relying on 10 country-specific indicators representing demographic, geographic and population-level health conditions (Table S1 in **Online Supplementary Document**) to extrapolate the mortality burden of influenza to 193 countries [4]. We applied the Stage 2 methodology to each respiratory year separately. To maintain sufficient diversity in the set of countries used (see **Table 1** and Table S2 in **Online Supplementary Document**), we restricted the analysis to years for which we had 19 or more Stage 1 country rate estimates (similar to the 20 countries used for the 2009 pandemic extrapolation procedure [4]). We also excluded the 2009 pandemic season (years of inclusions were 2002-2008 and 2010-2011). Most Stage 1 countries contributed 5 or more data-years (the one exception was Uruguay with 3 years). To describe the variability in mortality estimates by influenza season, we present the seasonal ranges rather than 95% confidence intervals. To test whether the multiple imputation models had sufficient information to capture between-country differences in a statistically reliable manner, we calculated reliability coefficients for each year and age group, with a value of 0.8 or higher indicating high reliability [4].

As a sensitivity analysis to assess the stability of the global and regional estimates, we performed a ‘leave-one-out’ analysis, in which we removed each of the 31 Stage 1 countries one at a time [4]. To facilitate comparison of our results with those of the CDC study [2], we also conducted a sensitivity analysis in which we included subnational data from India (2 years of data) and Kenya (4 years of data for people <65 years of age only). We decided not to use these estimates in our main analysis because, unlike our other Stage 1 data, they represented less than 1% of the populations of these countries and death counts were obtained through verbal autopsy methods rather than certified death certificate coding (**Table 1**).

### Predictors of influenza mortality

We used mixed-effects generalized linear models to identify predictors of influenza-associated excess mortality and assess the role of circulating influenza virus type and subtype, population, socio-economic development, population health status, and time trends (see **Online Supplementary Document** for details). Regression models were run separately for each age group, and for the set of Stage 1 (n = 31 countries) and Stage 2 countries. To assess the role of circulating influenza subtype, we used the WHO FluNET database [5] to create a categorical index based on the regionally dominant influenza subtypes in each year, defined as the subtype representing >75% of influenza specimens (A(H3N2), A(H1N1), or mixed subtypes). As there was not enough information to assess subtype dominance by country, we used WHO regions to group countries. No season was dominated by influenza B during the study period.

To assess the role of population health and socio-economic status, we compiled standard country-specific indicators from the Institute for Health Metrics and Evaluation project, including the Healthcare Access and Quality Index (HAQI) [6], and the Socio-Demographic Index (SDI) [7]; both indicators have been used in multiple global disease burden studies eg, [6,8]. The HAQI is a measure of personal health care access and quality; it is constructed using mortality rates from 32 causes of death that are generally not fatal in the presence of effective medical care (ie, amenable mortality) [6]. The SDI is a measure of overall development, and includes information on income per person, average years of education and total fertility rate [7]. For context, we also compiled baseline total annual respiratory death rates from the Global Burden of Disease project, combining upper respiratory deaths, lower respiratory deaths, and chronic respiratory deaths [9].

In the mixed-effects regression models, we considered random effects for country, and fixed effects for other predictors such as subtype, HAQI, SDI, baseline respiratory death rates, year and region. Inclusion of a term for year allowed for modelling of putative time trends in influenza-associated respiratory mortality. Akaike’s information criterion (AIC) was used for model selection. When relevant, we repeated our analyses on the full sample of all 193 WHO countries and the 31 Stage 1 countries to check the consistency of findings. The Stage 1 estimates do not make any prior assumptions about the relationship between influenza-associated mortality and development as estimates are directly derived from vital statistics observations. Stage 2 estimates are based on an imputation method that do not make direct assumptions about this relationship, although 4 of the 10 indicators used for imputation purposes are related to socio-economic and health development.

## RESULTS

### Stage 1 and Stage 2

**Figure 1:**
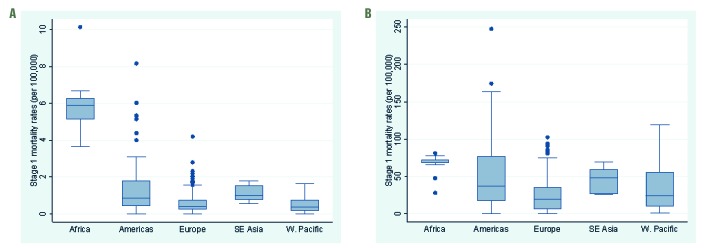
Boxplot of the Stage 1 country estimates of influenza-associated excess respiratory mortality rates per 100 000 by WHO region, under 65 and over 65. **Panel A.** Age <65. **Panel B.** Age ≥65.

We included Stage 1 estimates from 31 countries in our main analysis, representing 37% of the world population. Although three WHO regions (Europe, the Americas and Western Pacific) were well represented with 15, 8 and 8 countries, respectively, only one country estimate of influenza-associated excess respiratory death was available from Sub-Saharan Africa (South Africa) and South-East Asia (Thailand) each, and none from the Eastern Mediterranean WHO regions (**Table 1**). The Stage 1 country estimates differed by WHO region and age group, with wider inter-quartile ranges in the ≥65 age group compared to the <65 age group (**Figure 1**).

We estimated the average numbers and rates (per 100 000) of influenza-associated respiratory deaths globally, and by WHO region and age group (**Table 2**) in Stage 2. Overall, we estimated an annual mean of 389 000 influenza-associated respiratory deaths in the all-age group during the study period, with substantial annual variation, ranging from 294 000 deaths in 2002 to 518 000 in 2004 (**Table 2**). The magnitude of annual mortality fluctuations was quite similar for the less than and greater than 65 age groups (**Figure 3**). Nearly 50% of influenza-associated respiratory deaths occurred in South-East Asia and the Western Pacific and 67% occurred in individuals ≥65 years (**Table 1**). Age mortality patterns varied widely by WHO region, with the highest percentage of deaths in older individuals found in Europe (84%) and the lowest in Sub-Saharan Africa (36%). We found that the lowest percentage of deaths occurred in older adults in 2010, at the global level and in almost all regions (ie, the year after the 2009 pandemic).

**Table 2:** Average 2002-2011 (no 2009) seasonal influenza-associated excess mortality (numbers, rates per 100 000), by age group and WHO region

WHO region	Influenza-associated respiratory mortality estimates: numbers					Influenza-associated respiratory mortality estimates: rates per 100 000		
**Number, under 65 (range)**	**Number, 65+ (range)**	**Number, all ages (range)**	**Number of cases aged 65+ as percent of Total (range)**	**Geographical distribution of total (%)**	**Rates, under 65 (range)**	**Rates, 65+ (range)**	**Rates, all ages (range)**
Sub-Saharan Africa	27 530 (17 752-41 686)	15 565 (9446-21 079)	43 096 (31 312-62 765)	36% (28%-43%)	11%	3.7 (2.3-5.9)	65.0 (34.8-92.9)	5.6 (3.7-8.6)
Americas	13 743 (6802-22 175)	41 498 (17 572-66 604)	55 241 (26 707-88 779)	75% (63%-80%)	14%	1.7 (0.8-2.8)	54.9 (20.7-91.2)	6.2 (2.8-10.1)
Eastern Mediterranean	10 987 (8493-13 739)	12 732 (10 485-15 823)	23 719 (18 988-28 033)	54% (49%-57%)	6%	2.2 (1.7-2.8)	57.6 (43.6-77.1)	4.5 (3.5-5.6)
Europe	7687 (6568-9383)	39 064 (24 992-50 760)	46 751 (34 375-58 761)	84% (70%-86%)	12%	1.0 (0.9-1.2)	30.9 (19.1-40.8)	5.3 (3.8-6.7)
South-East Asia	39 747 (27 649-57 319)	62 665 (25 754-91 392)	101 411 (53 403-133 228)	61% (46%-69%)	26%	2.4 (1.7-3.6)	70.3 (33.7-103.2)	5.8 (3.3-7.6)
Western Pacific	26 031 (16 491-39 293)	63 605 (42 116-85 003)	89 637 (63 495-115 419)	71% (61%-79%)	23%	1.6 (1.0-2.5)	43.4 (29.6-55.3)	5.1 (3.7-6.1)
**World**	**128 512 (91 764-184 980)**	**260 701 (187 430-333 250)**	**389 213 (293 980-518 230)**	**67% (60%-72%)**	**100%**	**2.1 (1.6-3.1)**	**53.7 (35.6-71.7)**	**5.9 (4.0-8.0)**

WHO – World Health Organization

**Table 3:** Predictors of influenza-associated excess mortality rates per 100 000 by age group and country in the Stage 2 approach*

	Under 65 years, estimate (SE) significance†	Over 65 years, estimate (SE) significance†
**Intercept**	206.11 (13.34)‡	3644.71 (372.1)‡‡‡
**Health and socio-economic development:**		
-Healthcare And Quality Index (HAQI)§	-0.032 (0.004)‡‡‡	
-Socio-Demographic Index (SDI)‖	1.41 (0.35)‡‡‡	-18.47 (6.13)‡
-Baseline respiratory death rate¶	0.42 (0.09)‡‡‡	
**Viral characteristics:**		
-Mixed season	Ref	Ref
-Dominant A/H1N1	-0.14 (0.07)‡	3.04 (1.89)
-Dominant A/H1N1pdm	0.78 (0.08)‡‡‡	-0.63 (2.25)
-Dominant A/H3N2	0.51 (0.04)‡‡‡	9.13 (1.14)‡‡‡
**Region:**		
-Sub-Saharan Africa	Ref	Ref
-Eastern Mediterranean	-0.84 (0.11)‡‡‡	-2.09 (3.1)
-Europe	-1.87 (0.12)‡‡‡	-28.03 (3.35)‡‡‡
-Americas	-1.35 (0.11)‡‡‡	-3.56 (3.04)
-South-East Asia	-1.06 (0.13)‡‡‡	6.45 (3.74)
-Western Pacific	-1.72 (0.12)‡‡‡	-15.16 (3.35)‡‡‡
**Time trend:**		
-Year	-0.1 (0.01)‡‡‡	-1.78 (0.19)‡‡‡

SE – standard error

*Results of a multivariate mixed generalized linear regression model applied to estimated influenza-associated excess death rates in 193 countries over 9 y, 2002-2011, after exclusion of the pandemic period. Best model selected by AIC. The regression models explained 72% of the mortality variance in under 65 years and 38% in over 65 years.

†Significance level: ‡,<0.05; ‡‡,<0.001; ‡‡‡,<0.0001. We used the Kenward-Roger approximation to obtain approximate degrees of freedom for the mixed model, and the *t*-distribution for *P*-values.

§Healthcare And Quality Index (HAQI), reflecting amenable mortality causes (32 causes were considered). Higher values indicate higher health care access and quality [6].

‖Socio-Demographic Index (SDI), based on average income per person, educational attainment and the total fertility rate. SDI ranges between 0 and 1, with higher values indicating wealthier and more highly educated populations [7].

¶ Source: Institute for Health Metrics and Evaluation [9].

**Figure 2:**
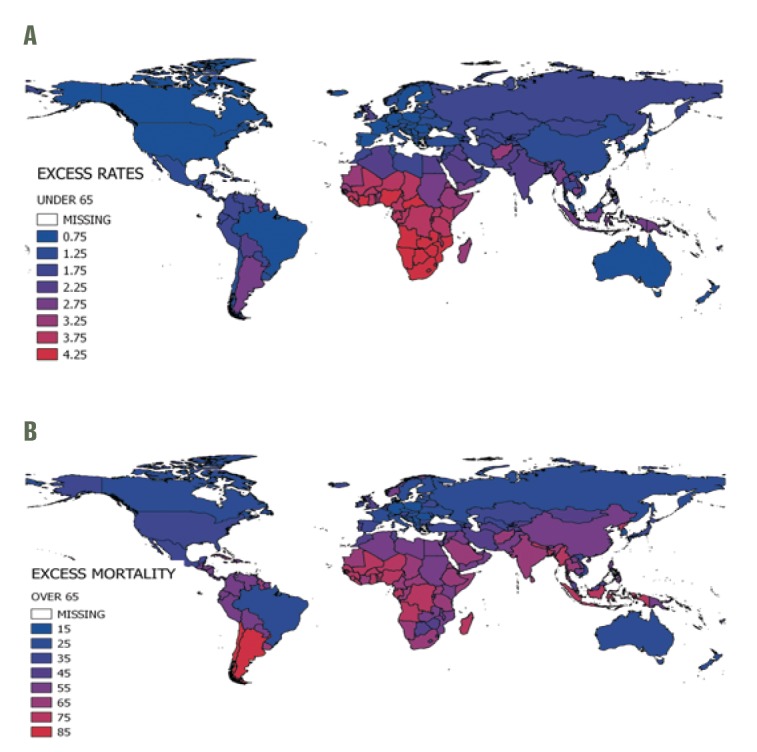
World map of average seasonal influenza-associated excess mortality rate per 100 000, by country*. **Panel A.** Age <65. **Panel B.** Age ≥65. *World map of average seasonal influenza-associated excess mortality rate per 100 000, by country.

The mean annual global influenza-associated respiratory mortality rate per 100 000 was 5.9, with regional estimates ranging from 4.5 in Eastern Mediterranean to 6.2 in the Americas (**Table 2**). Globally, the excess mortality rate was on average 26 times higher in older adults than in the <65 age group (ranging from 18 times higher in Sub-Saharan Africa to 32 times higher in the Americas). The highest estimated rates were in the <65 age group in Sub-Saharan Africa and the ≥65 group in South-East Asia. Some countries experienced particularly high mortality rates in the ≥65 group, including those in the Americas (eg, Argentina), Sub-Saharan Africa, the Middle East, South-East Asia and parts of Western Pacific (eg, China **Figure 2**, panel B). The WHO European region had the lowest influenza-associated respiratory mortality rates in both the <65 and ≥65 age groups.

**Figure 4:**
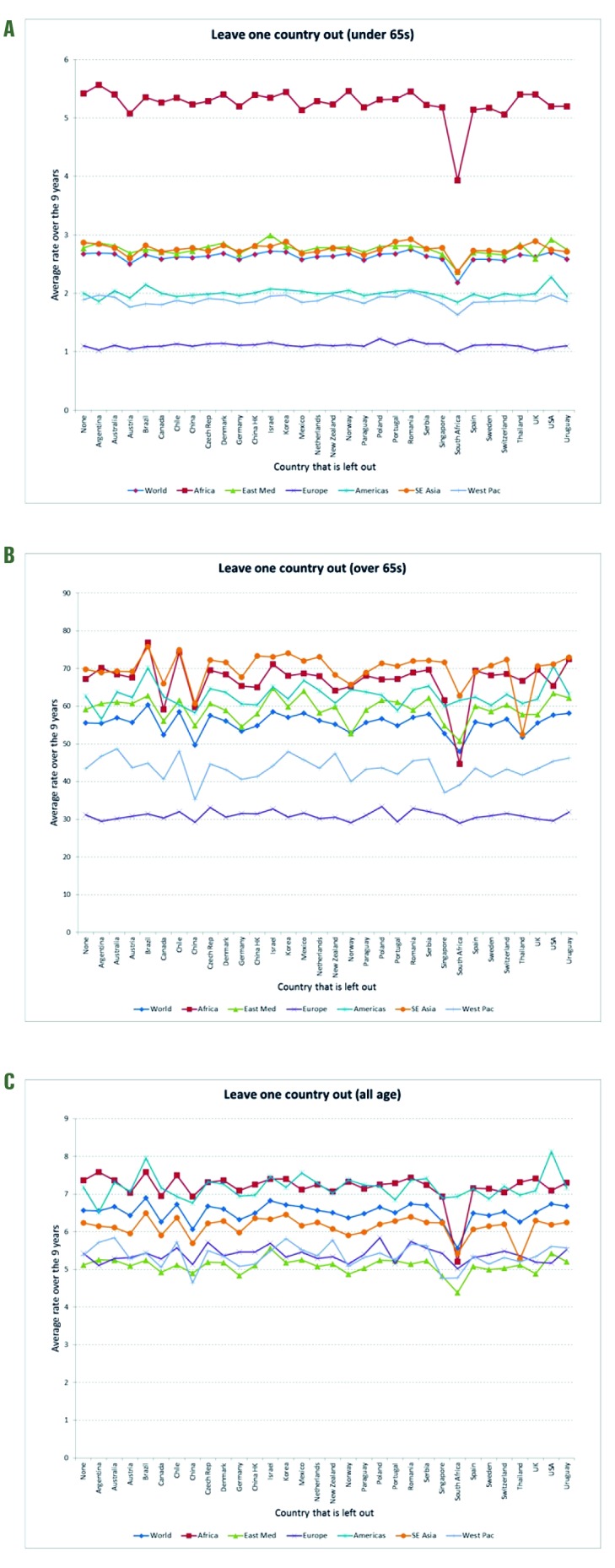
Sensitivity analysis of global and regional influenza–associated respiratory mortality rates per 100 000 population. **Panel A.** Age <65. **Panel B.** Age ≥65. **Panel C.** All ages.

**Sensitivity analyses.** To investigate the robustness of our global burden estimates, we conducted a sensitivity analysis which included the subnational data from smaller samples available in India (2 years) and Kenya (4 years; only <65 years age group). Including these data increased the global estimates **(****Figure 3**), especially in the <65 age group (Panel A). A ‘leave-one-out’ analysis of the Stage 1 estimates [7] (**Figure 4**) revealed that the influenza-associated respiratory mortality estimates were generally stable, but that South Africa (the only Stage 1 estimate for the African region in our main analysis) had an important impact on the global estimate and some of the regional estimates (eg, the sub-Saharan Africa and Eastern Mediterranean regions). The reliability coefficients for the nine annual models averaged 0.8 in the two age groups, indicating high reliability/internal consistency (see Table S3 in **Online Supplementary Document**).

### Predictors of influenza-associated respiratory mortality

Multivariate regression (**Table 3**) revealed that the dominant subtype circulating each season was a predictor of excess respiratory mortality, with the most severe seasons coinciding with circulation of influenza A(H3N2) in older adults, and with A(H1N1)pdm09 pandemic virus in the younger age group, even outside of the 2009 pandemic season. The best model for the <65 age group included all three health and socio-economic indicators considered (HAQI, SDI and baseline respiratory mortality), indicating higher influenza burden in countries with less developed health care delivery and higher baseline respiratory mortality. In models for individuals aged ≥65 years, the only significant country-level predictor was SDI, suggesting that demographic- and socio-economic factors, rather than baseline health, drive influenza-associated respiratory mortality in this age group. The strong scaling of influenza burden with baseline respiratory mortality rates in people <65 years, but not in older adults ≥65 years of age, was consistent in Stage 1 and Stage 2 samples (Figures S2 and S3 in **Online Supplementary Document**).

Significant regional effects were found in all models, persisting beyond the effect of country-level predictors and regional subtype dominance. From the age-specific models we found a particularly high influenza-associated respiratory mortality rate for the <65 age group in Sub-Saharan Africa and for older individuals in South-East Asia (**Table 3**). In contrast, Europeans of all ages experienced lower influenza-associated respiratory mortality rates, even after controlling for socio-economic and demographic conditions experienced in this region.

**Figure 5:**
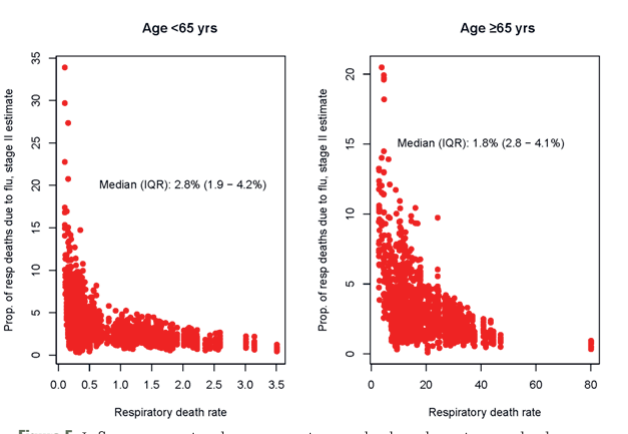
Influenza-associated excess respiratory death and respiratory death rates per 100 000 population (annual data).

To assess the overall influenza burden relative to other diseases associated with respiratory mortality, we explored the contribution of influenza-associated deaths to total respiratory deaths in our global sample. Overall, influenza-associated deaths represented a median 2.8% (IQR** = **1.9-4.2%) of total respiratory deaths in people <65 and 1.8% (IQR** = **2.8-4.1%) in those ≥65 years (**Figure 5**). The proportion of respiratory deaths attributable to influenza varied substantially between countries; however, these differences did not align with the socio-economic or health care indicators considered in this study. Estimates of the proportion of respiratory deaths due to influenza were consistent in Stage 1 (Figure S4 in the **Online Supplementary Document**) and Stage 2 data sets.

## DISCUSSION

Our study of global seasonal influenza-associated respiratory mortality is one of three influenza burden projects conducted in consultation with WHO; the others were led by the US-CDC [2] and GBD project [3]. We find that 389 000 deaths from respiratory causes are associated with influenza each year on average (range 294 000 - 518 000) during 2002-2011, excluding the 2009 pandemic season, implicating influenza in roughly two percent of all annual respiratory deaths. This estimate is similar to the CDC estimate – an important result in light of the very different global extrapolation methods used. Notably, the GLaMOR and CDC estimates are 2-3 fold higher than the GBD estimate. This is in part because the GLaMOR and CDC estimates include all influenza-associated respiratory deaths, while the GBD estimates only include deaths from lower respiratory tract infections that are directly caused by influenza; differences in extrapolation approaches for data-poor countries may also have played a role.

We further found that two-thirds (67%) of seasonal influenza deaths occurred in those ≥65 years of age but with large regional variation - from 36% in Sub-Saharan Africa to 86% in Europe; these differences are likely driven by regional variation in baseline mortality, age structure and socio-demographic development. Influenza-associated mortality rates were 26 times higher in those ≥65 years compared to those <65 years, highlighting the larger burden in the elderly and importance of this age group for mitigation of seasonal influenza.

Neither the GLaMOR nor CDC estimates captures influenza-associated deaths ascribed to cardiovascular causes, indicating that the total mortality burden of influenza is likely to be substantially higher. Had we analysed cardio-respiratory or all-cause mortality outcomes, our estimates would have had higher sensitivity (ie, captured more influenza-associated deaths) but would have had lower specificity (ie, had less precision). In the GLaMOR study of the 2009 influenza pandemic, that ratio of all-cause to respiratory influenza-associated mortality was about 2:1 [4]. A recent review of 43 influenza-related mortality studies [10] found that ratio varies considerably among countries because of demographic and population health differences such as frequency of chronic conditions; what the ratio might be on a global level is unknown.

We used regression analysis to investigate the factors associated with high influenza-associated respiratory mortality and found that mortality was largest in seasons dominated by A(H3N2) subtype in people over 65 years, but largest in seasons dominated by A(H1N1) in people 65 years and younger, in agreement with other country-specific studies [4,10,11]. Our subtype dominance assessment was based on regional data from FluNet, rather than country-specific information; sampling in FluNet can be limited, especially in earlier years, and may only include a few surveillance sites not representative of large countries, but it is a unique source of information available on a global scale. Further, we could not determine whether the increased A(H3N2) burden in older adults was due to higher attack rates, greater clinical severity (case fatality), or both. Further multi-national analyses of the variation in clinical severity by influenza subtype and strain would be useful, building on earlier work from Hong-Kong combining population-level data at different levels of the severity pyramid [12,13].

Regression analysis was further used to explore the relationship between influenza-associated mortality rates and indicators of development and health at the country level; this is important because prior global influenza studies made strong assumptions about this relationship [2,14]. We found that health care access [13], socio-demographic development indicators, and baseline respiratory mortality explained more than two-thirds of the variance in influenza-associated respiratory mortality rates between countries in people <65 years. These findings, if confirmed with additional observational data from low-income settings, suggest that improvements in health care might lower influenza-associated mortality in younger age groups. Given that high-income countries are near 100% on the HAQI scale of health development, the biggest gains in global influenza mortality would be expected in low and middle-income settings. In contrast, our regression model explained about a third of the variance in influenza-associated mortality in older individuals, on whom most of the burden of seasonal epidemics falls, with the socio-demographic indicator (SDI) being a significant predictor. This suggests that influenza-related mortality among older individuals is less driven by health care development and more by demographics. Reassuringly, relationships between influenza-associated mortality, development, and demographics were consistent in Stage 1 and 2 data. Stage 1 data did not make any prior assumptions about these relationships as influenza estimates relied on direct modelling of vital statistics in data-rich countries, lending support to our findings. It is worth noting that our Stage 2 extrapolation approach included some covariates that increase with development (eg, physicians per capita and gross national income), so there is some circularity in our predictor analysis of Stage 2 data. Ultimately, these relationships should be tested with direct Stage 1 observations from a larger set of countries, especially in low-income regions. Finally, we did not have access to global influenza vaccination uptake data and thus could not include this factor in our predictor analysis. The effect of seasonal influenza vaccination and other factors such as population age structure and the prevalence of HIV and TB [15] deserve further study.

Our regression analysis suggests that general health care improvement may have less of an impact on influenza-associated mortality in older adults. These age differences should be interpreted with caution given ecological nature of this study and the collinearity of the health care and socio-demographic development indicators considered here. We speculate that chronic conditions associated with severe influenza outcomes late in life are harder to prevent or treat, possibly explaining our findings. Analyses of long-term changes in influenza-related mortality in a subset of countries with robust historical records may clarify this important question. And although we did not find evidence of nonlinear relationships between influenza excess mortality, HAQI, and SDI, as important mortality causes shift with development, further analyses with more data points would be warranted.

The CDC-led study [2] and our study relied on similar data but were methodologically distinct. Both used an overlapping set of Stage 1 estimates from data-rich countries that had detailed vital statistics information, although we did not use data from India and Kenya while including data from Sweden, Poland and Brazil. The modelling approaches used to extrapolate to the regional and global levels, however, were quite different. In line with the GLaMOR approach [4], we imputed influenza mortality in countries without national vital statistics on the basis of 10 demographic, geographic, social and other indicators. In contrast, the US CDC-led study [2] used a “multiplier” method whereby influenza mortality is assumed to scale with age- and region-specific baseline respiratory mortality, modified from Dawood et al. [16]. As previously discussed, validation of this extrapolation approach requires careful investigation of the relationship between influenza associated-mortality and baseline respiratory mortality.

While the two methods produced broadly similar global burden estimates, that similarity obscures important differences. The CDC study found that 42% of deaths occurred in people <65 years of age, while we found that only 33% (range 28%-40%) in this age group. The CDC study found the highest burden in Sub-Saharan Africa while we found the highest burden in the Americas. Additional Stage 1 data from low-income settings would be needed to settle these differences.

Sensitivity analyses indicated that our estimates increased markedly when we included the Stage 1 inputs from India and Kenya that were included in the CDC study (**Figure 3**), which might explain why the CDC estimated proportion of deaths in people <65 years was higher than ours. We also note that the set of countries both teams used is a convenience sample, with substantial gaps. Indeed, we had no Stage 1 countries representing the Eastern Mediterranean and just one for each Sub-Saharan Africa and South-East Asia. For example, removing the South Africa Stage 1 estimate substantially decreased the mortality estimate for all of Sub-Saharan Africa (**Figure 4**); it is difficult, however, to assess the estimation error due to the lack of balanced geographic representation. Obviously, having more countries from each region would improve the mortality estimates from both studies, but that must await the collection of detailed vital statistics in more low-income countries

A recent review of Stage 1 mortality studies suggested that models that do not consider viral activity terms tend to overestimate the burden [10], and simulation analyses have shown that less robust surveillance tends to underestimate it [17]. These observations might explain why our Brazil estimate is so different from the other South American Stage 1 countries, which relied on the Serfling method that does not include viral terms (**Figure 2**).

The 2017 GBD mortality estimates [3] were lower (the point estimate was 145 000 deaths in all ages) than the GLaMOR and CDC estimates but this was to be expected as the GBD estimates were based on an assessment of lower respiratory tract infection deaths rather than all respiratory deaths (which include lower and upper respiratory tract deaths). Further, the GBD approach considers influenza ‘attributable’ (caused by) deaths rather than influenza associated-deaths, and would exclude, for example, a death where pneumonia is the principal cause of death and influenza was the secondary cause. Furthermore, the country-specific data from GBD indicates the highest morality burdens were found in eastern Europe (eg, Russia and Ukraine) and countries in Sub-Saharan Africa and South America [3]. The latter findings are generally consistent with the GLaMOR and US CDC estimates, but the very high rates in eastern Europe are not (see **Figure 2**), suggesting some important methodological differences in the calculation methods (eg, local or national studies could weigh substantially on the three population attributable factions used in the GBD method) [3].

## CONCLUSIONS

Going forward, proper monitoring of influenza-associated mortality requires the collection of more detailed mortality data from a larger set of countries and over a longer time period. As a case in point, while 70 countries report annual vital statistics to WHO [18], only 33 provided data at the weekly or monthly resolution needed for influenza Stage 1 burden studies. We believe most of the 70 participating countries have more detailed data at monthly or weekly time scale which, if reported and shared, would be very valuable in assessing any diseases that exhibits seasonality.

Our study highlights systematic regional variation in influenza mortality burden, in part driven by health care and socio-economic development, which should be further investigated as more data become available. Because estimation of influenza mortality is not straightforward and entails assumptions that are difficult to test, it is important to compare estimates from different modelling approaches, as we have done here. Further, our global and regional estimates will provide a useful baseline to set health priorities [19] and project the impact of new or improved intervention measures, such as universal influenza vaccines currently under development.

## Additional material

Online Supplementary Document

## 

**Table:** **Figure 3.** Yearly influenza-associated respiratory mortality rate per 100 000 population, with and without adding India and Kenya to the Stage 1 input data set. **Panel A.** Age <65. **Panel B.** Age ≥65. **Panel C.** All ages.
