# Supplementary material for: Global mortality associated with seasonal influenza epidemics: New burden estimates and predictors from the GLaMOR Project
Source: J Glob Health. 2019 Oct 22;9(2):020421. doi: 10.7189/jogh.09.020421 (PMC6815659; doi:10.7189/jogh.09.020421)

Supplementary Text and Results

Appendix S1. Supplementary Methods

Stage 1 Country-specific Influenza-associated Mortality Estimates. We first obtained estimates from 30 main countries using published data from the recent study by Iuliano et al [1] and data from three additional countries (Brazil, Sweden and Poland). All countries in the main analysis provided national vital statistics on respiratory mortality and most provided viral surveillance data (Main text Table 1). Because estimates from India and Kenya were based on verbal autopsy assessment of respiratory cause of death for a small or regional population sub-sample (1% of the total population in India and the Western Region of Kenya) [2], we elected to exclude both from the main analysis but used them in a sensitivity analysis to gauge the robustness of the global estimate. The main analysis is therefore based on 31 countries (Main text Table 1).

The modelling methods used to calculate influenza-associated mortality varied, with 26 of the 33 countries performing regression models with viral activity and seasonal terms as covariates, while the remaining 7 countries used Serfling models with seasonal terms only (reference [1] and Main text Table 1). Of the 7 countries using Serfling, 5 were in South America. Different seasonal terms were included in the models: most models included harmonic terms although a few used flexible spline functions; some also added temperature and humidity covariates ([1], Supplementary appendix, Appendix Table 2).

For Brazil, Poland and Sweden, we used negative binomial regression models driven by weekly viral activity, using cubic splines (5 or 6 degrees of freedom, depending on the country) to model seasonal baselines. The degree of freedom for the spline function was chosen based on minimization of auto-correlation. Viral activity indicators were based on the weekly percent positive for total influenza positive specimens reported to WHO FluNet and smoothed by a 3-wk average centered in the current week. Separate influenza coefficients were fit for separate influenza seasons.

Stage 2: Multiple Imputation. The method to extrapolate the global burden of influenza is based on a subset of world countries that have influenza-associated mortality estimates (Stage 1 sample) and has been described in detail elsewhere [5]. It involves two steps, a data creation step followed by a hierarchical regression modelling step to project the Stage 1 influenza burden estimates to 193 world countries. In the data creation step, we used statistical correlations between a set of country indicators (Table S1) and the Stage 1 mortality estimates to create a distribution of possible mortality

values for each of the countries without data. In the regression step, we applied a hierarchical linear random effects regression model to these distributions per country, to obtain a point estimate (with standard error) for the influenza burden estimates by country, region and the world. We provide more details on this approach in the next section.

We applied the Stage 2 methodology to data for each year separately, which ran from July 1 to June 30 for the Northern hemisphere and the actual calendar year for countries in the Southern Hemisphere or countries with tropical climates (Main text Table 1). We only included years for which we had 19 or more Stage 1 country estimates: specifically, the years 2002-2008 and 2010-2011, with 2009 pandemic year excluded (Table S2). To prepare the Stage 1 data for Stage 2 modelling, we replaced negative excess mortality estimates with zero and excluded country estimates for which the point estimate and confidence intervals were all zero (this only happened for the Netherlands).

Stage 2 Point Estimate Generation Procedure. To estimate global, regional and country-specific excess mortality rates using the created datasets (20 imputed data-points per country per year), we applied a hierarchical linear random effects regression model. For each year, we calculated the mortality rates simultaneously (in one model) for each country, region, and the world. Separate models were fitted for each year.

The model used was:

$$Y_{ij} = \beta_0 + \sum_{r=1}^{r} \beta_r X_r + \sum_{f=1}^{n-1} \beta_f X_f + \mu_j + \sum_{f=1}^{n} \varepsilon_f$$

Where:

Y = imputed individual measurement rates per country

i = individual measurement (1 to 20 for imputation)

j = country (1 …. 193)

μ_j = between country variance; $\mu_j \sim N(0,\tau_j^2)$

εf = error variance for every imputed dataset, normally distributed; $\varepsilon f \sim N(0, \tau_f^2)$

Xr = dummy variable for the six WHO regions (r=1…6), rescaled indicators ((0,1)-1/6)

and is 0 if r=7

r=7, countries not belonging to a region (1 if r=7 else 0)

r = WHO region (1..7)

Xf = dataset indicator, rescaled indicator coding ((0,1)-1/n)

f = imputed datasets (1..20)

n=number of datasets (20)

To estimate the different rates as predictions based on the model:

World = $\beta 0$

WHO Region = $\beta 0 + \sum \beta r$

Country = $\beta 0 + \sum \beta r + \mu_j$

We performed the imputation procedures with the Amelia software package [3], and used the MLwiN v2.3 package for the analysis model [4].

Stage 2 model outputs. Table S3 presents the results from the hierarchical linear random effects regression model for over 65 age and under 65 years models by year (2002-2008 and 2010-2011).

Appendix S2. Supplementary Results

Stage 1 sensitivity analyses

We compared the Stage 1 estimates from our 31 initial countries to their corresponding Stage 2 projected value and found excellent agreement (pairwise correlation >0.95, median difference 17% in under 65 years and 15% in 65 years & above, Figure S1).

Additional GLM findings

Time trends. We found significant declining time trends in influenza-associated Stage 2 mortality estimates in both age groups between 2002 and 2011, equivalent to a 3.5% (95% CI 2.8 - 4.2%) annual decline in the >65 year old age group and a 4.8% (4.1 – 5.4%) decline in the <65 year age group (Table S3). However, we saw no evidence of time trends in the smaller sample of 31 Stage 1 countries.

Interpreting the time trends is difficult. Because of the short time period and the pandemic interruption in 2009, the trends finding could merely be an artefact short-term perturbations in influenza circulation in the peri-pandemic period; indeed, the beginning of our study period coincided with severe epidemics caused by antigenically novel A/H3N2 viruses. Alternatively, these declining trends could reflect long-term improvements in healthcare not specific to influenza, or perhaps increasing influenza vaccination or drug treatment. The effect of influenza vaccination certainly deserves further study as more data years become available.

Other factors. Additional analyses to identify predictors of excess mortality in Stage 1 data as in were consistent with our main analysis using Stage 2 estimates, and pointed at subtype and regional effects, as well as health and socio-economic indicators (Table S3). These results confirm the importance of country-specific development indicators in driving influenza-related mortality, although the power of this analysis is limited by the lack of less developed countries in the Stage1 sample.

Supplementary References

1 Iuliano AD, Roguski KM, Chang HH, Muscatello DJ, Palekar R, Tempia S, et al. Estimates of global seasonal influenza-associated respiratory mortality: a modelling study. Lancet. 2018; 391:1285-1300.

2 Emukule GO, Spreeuwenberg P, Chaves SS, Mott JA, Tempia S, Bigogo G, et al. Estimating influenza and respiratory syncytial virus-associated mortality in Western Kenya using health and demographic surveillance system data, 2007-2013. PLoS One. 2017:12:e0180890.

3. Honaker J, King G, Blackwell M. AMELIA II : A Program for Missing Data. J Stat Softw. 2011;45:1–54. Available from: http://gking.harvard.edu/amelia/

4 Rabash J, Charlton C, Browne W, Healy M, Cameron B. A User's Guide to MLwiN Version 2.30. University of Bristol; 2009.

5 Simonsen L, Spreeuwenberg P, Lustig R, Taylor RJ, Fleming DM, Kroneman M, et al. Global Mortality Estimates for the 2009 Influenza Pandemic from the GLaMOR Project: A Modeling Study. PLoS Med. 2013;10:e1001558.

SUPPLEMENTARY TABLES

Table S1. Country indicators used as factors in the Stage 2 model [5] that projects the measured Stage 1 seasonal influenza mortality.

Indicator number	Indicator
1	WHO region (Africa, Americas, Eastrn Mediterranean, Europe, South-East Asia, Western Pacific)
2	Age group all cause mortality rates (0-14-15-64, 65+)
3	Physician density (per 10,000 population)
4	Obesity (percent with body mass index >30kg/m ^{2}
5	Population density (per km ^{2})
6	Major infectious diseases (percent HIV positive and tuberculosis prevalence)
7	Gross National Income (GNI) per capita (US dollars)
8	Rural population (percent)
9	Population age structure: percent <15 and >60 years
10	Latitude (absolute value)

Table S2. Stage 1 country included in each seasonal estimate by year and WHO region.

**Indicator number**	2001	2002	2003	2004	2005	2006	2007	2008	2009	2010	2011	2012
Countries	Argentina	Argentina	Argentina	Argentina	Argentina	Argentina	Argentina	Argentina	Argentina			
			Australia	Australia	Australia	Australia	Australia	Australia	Australia			
	Austria	Austria	Austria	Austria	Austria	Austria	Austria	Austria	Austria			
				Brazil	Brazil	Brazil	Brazil	Brazil	Brazil	Brazil	Brazil	Brazil
	Canada	Canada	Canada	Canada	Canada	Canada	Canada					
		Chile	Chile	Chile	Chile	Chile	Chile	Chile	Chile			
				China	China	China	China	China	China			
	Czech Republic	Czech Republic	Czech Republic	Czech Republic	Czech Republic	Czech Republic	Czech Republic	Czech Republic	Czech Republic	Czech Republic		
		Denmark	Denmark	Denmark	Denmark	Denmark	Denmark	Denmark	Denmark	Denmark	Denmark	Denmark
						England&Wales	England&Wales	England&Wales	England&Wales	England&Wales	England&Wales	
	Germany	Germany	Germany	Germany	Germany	Germany	Germany	Germany	Germany	Germany	Germany	
	Hong Kong	Hong Kong	Hong Kong	Hong Kong	Hong Kong	Hong Kong	Hong Kong	Hong Kong	Hong Kong	Hong Kong	Hong Kong	
				Israel	Israel	Israel	Israel	Israel	Israel	Israel	Israel	
		Mexico	Mexico	Mexico	Mexico	Mexico	Mexico	Mexico	Mexico			
	Netherlands	Netherlands	Netherlands	Netherlands	Netherlands	Netherlands	Netherlands	Netherlands	Netherlands	Netherlands		
		New Zealand	New Zealand	New Zealand	New Zealand	New Zealand	New Zealand	New Zealand	New Zealand	New Zealand	New Zealand	
	Norway	Norway	Norway	Norway	Norway	Norway	Norway	Norway	Norway	Norway	Norway	
		Paraguay	Paraguay	Paraguay	Paraguay	Paraguay	Paraguay	Paraguay	Paraguay			
		Poland	Poland	Poland	Poland	Poland	Poland	Poland	Poland	Poland	Poland	Poland
	Portugal	Portugal	Portugal	Portugal		Portugal	Portugal	Portugal	Portugal	Portugal	Portugal	
	Romania	Romania	Romania	Romania	Romania	Romania	Romania	Romania	Romania	Romania	Romania	
	Serbia	Serbia	Serbia	Serbia	Serbia	Serbia	Serbia	Serbia	Serbia	Serbia		
				Singapore	Singapore	Singapore	Singapore	Singapore		Singapore	Singapore	
	South Africa	South Africa	South Africa	South Africa	South Africa	South Africa	South Africa	South Africa	South Africa	South Africa	South Africa	South Africa
			South Korea	South Korea	South Korea	South Korea	South Korea	South Korea	South Korea	South Korea	South Korea	
	Spain	Spain	Spain	Spain	Spain	Spain	Spain	Spain	Spain	Spain	Spain	Spain
			Sweden	Sweden	Sweden	Sweden	Sweden	Sweden	Sweden	Sweden	Sweden	
	Switzerland	Switzerland	Switzerland	Switzerland	Switzerland	Switzerland	Switzerland	Switzerland	Switzerland	Switzerland	Switzerland	Switzerland
						Thailand	Thailand	Thailand	Thailand	Thailand	Thailand	
				Uruguay			Uruguay	Uruguay	Uruguay			
	USA	USA	USA	USA	USA	USA	USA	USA	USA	USA	USA	USA
Number of countries	15	**21**	**24**	**29**	**27**	**30**	**31**	**30**	**29**	**22**	**19**	7
Number of WHO regions	4	**4**	**4**	**4**	**4**	**5**	**5**	**5**	**5**	**5**	**5**	3
Sensitivity analysis:										India	India	India
							Kenya (<65)	Kenya (<65)	Kenya (<65)	Kenya (<65)	Kenya (<65)	Kenya (<65)

Table S3. Stage 2 model outputs by year (2002-2011) and age group (over 65 and under 65) - Results from the hierarchical linear random effects regression model *

**Year 2002**	**OVER 65**		**UNDER 65**	
**Fixed effects**	**Estimate (standard error)**	**P value**	**Estimate (standard error)**	**P value**
Intercept world	42.41 (1.15)	0.00	1.824 (0.046)	0.000
Region (rest)	15.78 (4.71)	0.00	-0.078 (0.187)	0.678
Region Africa	30.82 (3.63)	0.00	2.108 (0.144)	0.000
Region east-med	20.21 (4.22)	0.00	0.452 (0.167)	0.007
Region Europe	-5.01 (3.58)	0.16	-0.381 (0.142)	0.007
Region America	12.72 (3.85)	0.00	0.179 (0.153)	0.242
Region SEAR	1.31 (5.08)	0.80	0.424 (0.201)	0.035
Factor 2	-0.55 (1.64)	0.74	0.165 (0.094)	0.078
Factor 3	1.13 (1.69)	0.51	-0.022 (0.091)	0.810
Factor 4	-1.55 (1.66)	0.35	0.054 (0.092)	0.559
Factor 5	-1.33 (1.62)	0.41	-0.041 (0.092)	0.652
Factor 6	-0.87 (1.66)	0.60	0.025 (0.092)	0.785
Factor 7	-1.32 (1.64)	0.42	-0.096 (0.090)	0.284
Factor 8	1.59 (1.64)	0.33	-0.068 (0.090)	0.454
Factor 9	-1.77 (1.63)	0.28	0.112 (0.095)	0.237
Factor 10	-1.96 (1.60)	0.22	0.130 (0.094)	0.165
Factor 11	0.68 (1.61)	0.67	0.104 (0.094)	0.270
Factor 12	-1.39 (1.64)	0.40	0.011 (0.094)	0.907
Factor 13	0.26 (1.67)	0.88	0.116 (0.093)	0.210
Factor 14	0.62 (1.63)	0.71	0.140 (0.098)	0.155
Factor 15	-0.16 (1.63)	0.92	-0.026 (0.093)	0.776
Factor 16	-1.43 (1.59)	0.37	0.051 (0.093)	0.584
Factor 17	-1.49 (1.62)	0.36	0.094 (0.096)	0.326
Factor 18	0.87 (1.66)	0.60	-0.008 (0.089)	0.931
Factor 19	-1.36 (1.64)	0.41	-0.021 (0.092)	0.816
Factor 20	-0.32 (1.64)	0.85	0.032 (0.094)	0.735

**Random effects**	**Variance(standard error)**	**P value**	**Variance(standard error)**	**P value**
Between countries	170.31 (18.75)	*0.000*	0.245 (0.029)	*0.000*
Factor 1 error	804.23 (47.94)	*0.000*	2.445 (0.146)	*0.000*
Factor 2 error	746.67 (44.57)	*0.000*	2.664 (0.159)	*0.000*
Factor 3 error	847.28 (50.48)	*0.000*	2.386 (0.142)	*0.000*
Factor 4 error	796.54 (47.50)	*0.000*	2.471 (0.147)	*0.000*
Factor 5 error	723.50 (43.22)	*0.000*	2.421 (0.144)	*0.000*
Factor 6 error	798.29 (47.60)	*0.000*	2.456 (0.146)	*0.000*
Factor 7 error	753.39 (44.96)	*0.000*	2.206 (0.132)	*0.000*
Factor 8 error	750.00 (44.77)	*0.000*	2.274 (0.136)	*0.000*
Factor 9 error	726.54 (43.40)	*0.000*	2.772 (0.165)	*0.000*
Factor 10 error	674.65 (40.32)	*0.000*	2.655 (0.158)	*0.000*
Factor 11 error	694.34 (41.50)	*0.000*	2.666 (0.159)	*0.000*
Factor 12 error	755.69 (45.09)	*0.000*	2.651 (0.158)	*0.000*
Factor 13 error	812.76 (48.45)	*0.000*	2.528 (0.151)	*0.000*
Factor 14 error	738.58 (44.08)	*0.000*	3.165 (0.188)	*0.000*
Factor 15 error	739.16 (44.12)	*0.000*	2.534 (0.151)	*0.000*
Factor 16 error	666.19 (39.85)	*0.000*	2.599 (0.155)	*0.000*
Factor 17 error	720.17 (42.99)	*0.000*	2.842 (0.169)	*0.000*
Factor 18 error	781.66 (46.64)	*0.000*	2.132 (0.127)	*0.000*
Factor 19 error	752.00 (44.89)	*0.000*	2.488 (0.148)	*0.000*
Factor 20 error	753.28 (44.97)	*0.000*	2.664 (0.159)	*0.000*
**Reliability**	0.833		0.679	

**Year 2003**	**OVER 65**		**UNDER 65**	
**Fixed effects**	**Estimate (standard error)**	**P value**	**Estimate (standard error)**	**P value**
Intercept world	61.16 (1.53)	*0.00*	2.546 (0.071)	*0.000*
Region (rest)	19.64 (6.25)	*0.00*	-0.118 (0.290)	*0.684*

Region Africa	42.27 (4.82)	*0.00*	2.928 (0.224)	*0.000*
Region east-med	40.31 (5.60)	*0.00*	0.812 (0.260)	*0.002*
Region Europe	-1.17 (4.74)	*0.80*	-0.667 (0.220)	*0.002*
Region America	50.67 (5.11)	*0.00*	0.894 (0.237)	*0.000*
Region SEAR	14.27 (6.73)	*0.03*	0.869 (0.312)	*0.005*
Factor 2	-0.78 (1.95)	*0.69*	0.071 (0.090)	*0.429*
Factor 3	-0.40 (2.00)	*0.84*	0.102 (0.091)	*0.265*
Factor 4	-1.46 (2.00)	*0.47*	0.029 (0.089)	*0.742*
Factor 5	0.07 (2.02)	*0.97*	0.032 (0.089)	*0.721*
Factor 6	-1.77 (1.96)	*0.37*	0.046 (0.090)	*0.607*
Factor 7	-0.11 (2.00)	*0.96*	-0.023 (0.089)	*0.797*
Factor 8	-2.06 (1.93)	*0.29*	0.120 (0.090)	*0.181*
Factor 9	-1.42 (1.99)	*0.47*	0.068 (0.092)	*0.459*
Factor 10	-0.80 (1.94)	*0.68*	0.007 (0.091)	*0.935*
Factor 11	-0.11 (2.01)	*0.96*	0.069 (0.090)	*0.446*
Factor 12	-1.78 (2.01)	*0.37*	0.066 (0.091)	*0.469*
Factor 13	0.08 (1.96)	*0.97*	0.125 (0.091)	*0.166*
Factor 14	-1.87 (1.96)	*0.34*	0.016 (0.090)	*0.857*
Factor 15	-1.86 (1.96)	*0.34*	0.093 (0.088)	*0.292*
Factor 16	-0.86 (1.95)	*0.66*	0.075 (0.091)	*0.412*
Factor 17	1.09 (2.01)	*0.59*	0.067 (0.091)	*0.461*
Factor 18	-1.82 (1.96)	*0.35*	0.135 (0.092)	*0.144*
Factor 19	0.13 (1.95)	*0.95*	0.031 (0.090)	*0.728*
Factor 20	0.71 (1.99)	*0.72*	0.082 (0.091)	*0.366*
**Random effects**	**Variance(standard error)**	**P value**	**Variance(standard error)**	**P value**
Between countries	303.66 (32.97)	*0.000*	0.652 (0.071)	*0.000*
Factor 1 error	1187.20 (70.77)	*0.000*	2.268 (0.136)	*0.000*
Factor 2 error	1016.90 (60.77)	*0.000*	2.400 (0.143)	*0.000*
Factor 3 error	1132.80 (67.57)	*0.000*	2.535 (0.151)	*0.000*

Factor 4 error	1130.40 (67.43)	*0.000*	2.359 (0.141)	*0.000*
Factor 5 error	1178.10 (70.24)	*0.000*	2.347 (0.140)	*0.000*
Factor 6 error	1028.80 (61.48)	*0.000*	2.461 (0.147)	*0.000*
Factor 7 error	1126.20 (67.18)	*0.000*	2.339 (0.140)	*0.000*
Factor 8 error	963.22 (57.61)	*0.000*	2.407 (0.144)	*0.000*
Factor 9 error	1108.70 (66.14)	*0.000*	2.592 (0.155)	*0.000*
Factor 10 error	996.25 (59.56)	*0.000*	2.478 (0.148)	*0.000*
Factor 11 error	1152.20 (68.71)	*0.000*	2.473 (0.148)	*0.000*
Factor 12 error	1144.90 (68.29)	*0.000*	2.569 (0.153)	*0.000*
Factor 13 error	1032.90 (61.69)	*0.000*	2.487 (0.148)	*0.000*
Factor 14 error	1042.00 (62.21)	*0.000*	2.430 (0.145)	*0.000*
Factor 15 error	1032.50 (61.69)	*0.000*	2.257 (0.135)	*0.000*
Factor 16 error	1011.50 (60.45)	*0.000*	2.531 (0.151)	*0.000*
Factor 17 error	1141.60 (68.08)	*0.000*	2.510 (0.150)	*0.000*
Factor 18 error	1043.70 (62.34)	*0.000*	2.652 (0.158)	*0.000*
Factor 19 error	1003.90 (59.98)	*0.000*	2.435 (0.145)	*0.000*
Factor 20 error	1111.20 (66.30)	*0.000*	2.531 (0.151)	*0.000*
**Reliability**	0.861		0.854	

**Year 2004**	**OVER 65**		**UNDER 65**	
**Fixed effects**	**Estimate (standard error)**	**P value**	**Estimate (standard error)**	**P value**
Intercept world	71.75 (1.84)	*0.00*	3.096 (0.088)	*0.000*
Region (rest)	14.30 (7.53)	*0.06*	-0.465 (0.361)	*0.197*
Region Africa	37.64 (5.81)	*0.00*	3.426 (0.278)	*0.000*
Region east-med	12.35 (6.75)	*0.07*	0.296 (0.323)	*0.360*
Region Europe	-14.51 (5.72)	*0.01*	-1.412 (0.274)	*0.000*
Region America	35.96 (6.16)	*0.00*	0.283 (0.295)	*0.338*
Region SEAR	27.49 (8.11)	*0.00*	1.118 (0.388)	*0.004*
Factor 2	1.05 (3.56)	*1.00*	-0.023 (0.117)	*0.841*
Factor 3	-3.34 (3.34)	*0.77*	-0.002 (0.119)	*0.983*

Factor 4	-1.66 (3.41)	*0.32*	0.065 (0.119)	*0.585*
Factor 5	2.03 (3.51)	*0.63*	0.013 (0.123)	*0.915*
Factor 6	-1.76 (3.41)	*0.56*	-0.038 (0.121)	*0.751*
Factor 7	0.31 (3.51)	*0.61*	-0.005 (0.116)	*0.963*
Factor 8	1.07 (3.53)	*0.93*	0.025 (0.120)	*0.837*
Factor 9	-6.74 (3.35)	*0.76*	0.023 (0.116)	*0.843*
Factor 10	-0.09 (3.45)	*0.04*	0.010 (0.117)	*0.935*
Factor 11	-5.18 (3.47)	*0.98*	0.056 (0.117)	*0.633*
Factor 12	-2.54 (3.38)	*0.14*	0.074 (0.119)	*0.536*
Factor 13	-2.93 (3.37)	*0.45*	0.022 (0.120)	*0.854*
Factor 14	-2.95 (3.40)	*0.38*	0.104 (0.120)	*0.387*
Factor 15	-0.80 (3.45)	*0.38*	-0.061 (0.123)	*0.619*
Factor 16	-2.90 (3.41)	*0.82*	0.025 (0.118)	*0.830*
Factor 17	-2.05 (3.43)	*0.39*	0.004 (0.121)	*0.973*
Factor 18	-0.73 (3.42)	*0.55*	-0.060 (0.120)	*0.618*
Factor 19	-1.09 (3.48)	*0.83*	-0.030 (0.118)	*0.800*
Factor 20	-4.98 (3.44)	*0.75*	-0.084 (0.119)	*0.478*
**Random effects**	**Variance(standard error)**	**P value**	**Variance(standard error)**	**P value**
Between countries	410.76 (47.54)	*0.000*	0.999 (0.109)	*0.000*
Factor 1 error	3453.40 (205.87)	*0.000*	3.879 (0.232)	*0.000*
Factor 2 error	3875.60 (230.68)	*0.000*	3.994 (0.239)	*0.000*
Factor 3 error	3006.70 (179.62)	*0.000*	4.274 (0.255)	*0.000*
Factor 4 error	3274.80 (195.38)	*0.000*	4.359 (0.260)	*0.000*
Factor 5 error	3661.20 (218.09)	*0.000*	4.829 (0.288)	*0.000*
Factor 6 error	3265.60 (194.83)	*0.000*	4.588 (0.274)	*0.000*
Factor 7 error	3690.40 (219.80)	*0.000*	3.928 (0.235)	*0.000*
Factor 8 error	3750.60 (223.34)	*0.000*	4.439 (0.265)	*0.000*
Factor 9 error	3025.50 (180.72)	*0.000*	3.869 (0.231)	*0.000*
Factor 10 error	3456.30 (206.04)	*0.000*	4.010 (0.240)	*0.000*

Factor 11 error	3500.80 (208.66)	*0.000*	4.021 (0.240)	*0.000*
Factor 12 error	3167.50 (189.07)	*0.000*	4.370 (0.261)	*0.000*
Factor 13 error	3121.30 (186.36)	*0.000*	4.404 (0.263)	*0.000*
Factor 14 error	3241.50 (193.42)	*0.000*	4.422 (0.264)	*0.000*
Factor 15 error	3429.20 (204.45)	*0.000*	4.943 (0.294)	*0.000*
Factor 16 error	3264.30 (194.76)	*0.000*	4.176 (0.249)	*0.000*
Factor 17 error	3341.30 (199.28)	*0.000*	4.618 (0.275)	*0.000*
Factor 18 error	3306.40 (197.23)	*0.000*	4.524 (0.270)	*0.000*
Factor 19 error	3542.80 (211.13)	*0.000*	4.126 (0.246)	*0.000*
Factor 20 error	3390.70 (202.19)	*0.000*	4.288 (0.256)	*0.000*
**Reliability**	0.728		0.834	

**Year 2005**	**OVER 65**		**UNDER 65**	
**Fixed effects**	**Estimate (standard error)**	**P value**	**Estimate (standard error)**	**P value**
Intercept world	58.65 (1.52)	*0.00*	2.233 (0.061)	*0.000*
Region (rest)	3.35 (6.22)	*0.59*	-0.310 (0.249)	*0.214*
Region Africa	25.03 (4.80)	*0.00*	2.347 (0.192)	*0.000*
Region east-med	6.34 (5.57)	*0.26*	0.256 (0.223)	*0.252*
Region Europe	-30.01 (4.72)	*0.00*	-0.986 (0.189)	*0.000*
Region America	12.67 (5.09)	*0.01*	0.050 (0.204)	*0.806*
Region SEAR	13.93 (6.70)	*0.04*	0.575 (0.268)	*0.032*
Factor 2	0.36 (2.55)	*0.89*	-0.113 (0.087)	*0.195*
Factor 3	0.08 (2.61)	*0.97*	-0.084 (0.087)	*0.333*
Factor 4	-3.41 (2.51)	*0.17*	0.010 (0.089)	*0.910*
Factor 5	-1.48 (2.61)	*0.57*	0.003 (0.090)	*0.976*
Factor 6	0.26 (2.57)	*0.92*	-0.058 (0.089)	*0.511*
Factor 7	-0.93 (2.49)	*0.71*	-0.040 (0.090)	*0.659*
Factor 8	1.68 (2.67)	*0.53*	-0.092 (0.086)	*0.283*
Factor 9	1.22 (2.59)	*0.64*	-0.087 (0.090)	*0.330*
Factor 10	-0.49 (2.57)	*0.85*	-0.048 (0.091)	*0.596*

Factor 11	-1.58 (2.61)	*0.54*	0.008 (0.090)	*0.928*
Factor 12	-1.13 (2.55)	*0.66*	-0.051 (0.088)	*0.559*
Factor 13	-0.11 (2.58)	*0.97*	-0.066 (0.087)	*0.447*
Factor 14	-1.17 (2.53)	*0.64*	-0.110 (0.088)	*0.211*
Factor 15	-0.65 (2.62)	*0.80*	-0.006 (0.089)	*0.948*
Factor 16	1.39 (2.59)	*0.59*	-0.070 (0.088)	*0.426*
Factor 17	-0.23 (2.59)	*0.93*	-0.055 (0.088)	*0.535*
Factor 18	0.08 (2.56)	*0.98*	-0.035 (0.088)	*0.690*
Factor 19	0.71 (2.61)	*0.78*	-0.102 (0.088)	*0.250*
Factor 20	-2.74 (2.48)	*0.27*	-0.116 (0.089)	*0.193*
**Random effects**	**Variance(standard error)**	**P value**	**Variance(standard error)**	**P value**
Between countries	286.41 (32.41)	*0.000*	0.474 (0.052)	*0.000*
Factor 1 error	1903.70 (113.58)	*0.000*	2.321 (0.138)	*0.000*
Factor 2 error	1858.00 (110.89)	*0.000*	2.096 (0.125)	*0.000*
Factor 3 error	2043.80 (121.81)	*0.000*	2.056 (0.123)	*0.000*
Factor 4 error	1732.40 (103.51)	*0.000*	2.310 (0.138)	*0.000*
Factor 5 error	2040.70 (121.63)	*0.000*	2.406 (0.143)	*0.000*
Factor 6 error	1906.40 (113.73)	*0.000*	2.241 (0.134)	*0.000*
Factor 7 error	1699.60 (101.58)	*0.000*	2.369 (0.141)	*0.000*
Factor 8 error	2209.00 (131.52)	*0.000*	1.918 (0.115)	*0.000*
Factor 9 error	1976.20 (117.84)	*0.000*	2.330 (0.139)	*0.000*
Factor 10 error	1910.30 (113.96)	*0.000*	2.505 (0.149)	*0.000*
Factor 11 error	2029.50 (120.98)	*0.000*	2.339 (0.139)	*0.000*
Factor 12 error	1855.30 (110.73)	*0.000*	2.123 (0.127)	*0.000*
Factor 13 error	1940.50 (115.74)	*0.000*	2.108 (0.126)	*0.000*
Factor 14 error	1811.50 (108.16)	*0.000*	2.187 (0.131)	*0.000*
Factor 15 error	2065.80 (123.10)	*0.000*	2.225 (0.133)	*0.000*
Factor 16 error	1972.00 (117.59)	*0.000*	2.141 (0.128)	*0.000*
Factor 17 error	1989.40 (118.61)	*0.000*	2.164 (0.129)	*0.000*

Factor 18 error	1896.90 (113.18)	*0.000*	2.168 (0.129)	*0.000*
Factor 19 error	2037.00 (121.41)	*0.000*	2.198 (0.131)	*0.000*
Factor 20 error	1665.60 (99.59)	*0.000*	2.254 (0.134)	*0.000*
**Reliability**	0.766		0.825	

**Year 2006**	**OVER 65**		**UNDER 65**	
**Fixed effects**	**Estimate (standard error)**	**P value**	**Estimate (standard error)**	**P value**
Intercept world	50.59 (1.27)	*0.00*	2.208 (0.065)	*0.000*
Region (rest)	3.01 (5.20)	*0.56*	-0.244 (0.265)	*0.358*
Region Africa	19.44 (4.01)	*0.00*	2.235 (0.205)	*0.000*
Region east-med	16.89 (4.66)	*0.00*	0.853 (0.238)	*0.000*
Region Europe	-18.00 (3.95)	*0.00*	-0.895 (0.202)	*0.000*
Region America	-4.10 (4.25)	*0.34*	-0.419 (0.217)	*0.054*
Region SEAR	21.26 (5.60)	*0.00*	0.442 (0.286)	*0.122*
Factor 2	1.00 (2.61)	*0.70*	-0.097 (0.126)	*0.440*
Factor 3	1.31 (2.58)	*0.61*	-0.029 (0.121)	*0.813*
Factor 4	1.22 (2.63)	*0.64*	-0.115 (0.118)	*0.329*
Factor 5	-0.56 (2.54)	*0.83*	-0.053 (0.125)	*0.669*
Factor 6	0.07 (2.53)	*0.98*	-0.115 (0.118)	*0.329*
Factor 7	2.41 (2.64)	*0.36*	-0.050 (0.123)	*0.683*
Factor 8	-0.15 (2.50)	*0.95*	-0.179 (0.118)	*0.129*
Factor 9	2.04 (2.57)	*0.43*	-0.109 (0.119)	*0.363*
Factor 10	2.48 (2.66)	*0.35*	-0.109 (0.122)	*0.373*
Factor 11	2.56 (2.68)	*0.34*	-0.192 (0.117)	*0.102*
Factor 12	-0.99 (2.46)	*0.69*	0.013 (0.122)	*0.913*
Factor 13	1.04 (2.56)	*0.68*	-0.093 (0.118)	*0.428*
Factor 14	1.76 (2.61)	*0.50*	-0.099 (0.118)	*0.404*
Factor 15	-0.89 (2.51)	*0.72*	-0.120 (0.117)	*0.304*
Factor 16	2.40 (2.57)	*0.35*	-0.184 (0.118)	*0.117*
Factor 17	-0.39 (2.55)	*0.88*	-0.075 (0.122)	*0.541*

Factor 18	1.89 (2.57)	*0.46*	-0.138 (0.120)	*0.248*
Factor 19	1.09 (2.56)	*0.67*	-0.138 (0.120)	*0.251*
Factor 20	2.54 (2.62)	*0.33*	-0.251 (0.116)	*0.031*
**Random effects**	**Variance(standard error)**	**P value**	**Variance(standard error)**	**P value**
Between countries	190.97 (22.64)	*0.000*	0.515 (0.059)	*0.000*
Factor 1 error	1949.90 (116.19)	*0.000*	4.378 (0.261)	*0.000*
Factor 2 error	1992.30 (118.68)	*0.000*	4.802 (0.286)	*0.000*
Factor 3 error	1914.50 (114.11)	*0.000*	4.069 (0.243)	*0.000*
Factor 4 error	2045.00 (121.78)	*0.000*	3.678 (0.220)	*0.000*
Factor 5 error	1800.00 (107.38)	*0.000*	4.623 (0.275)	*0.000*
Factor 6 error	1741.80 (103.95)	*0.000*	3.640 (0.217)	*0.000*
Factor 7 error	2076.50 (123.63)	*0.000*	4.451 (0.265)	*0.000*
Factor 8 error	1675.00 (100.03)	*0.000*	3.703 (0.221)	*0.000*
Factor 9 error	1883.30 (112.27)	*0.000*	3.886 (0.232)	*0.000*
Factor 10 error	2139.20 (127.31)	*0.000*	4.250 (0.253)	*0.000*
Factor 11 error	2219.80 (132.05)	*0.000*	3.599 (0.215)	*0.000*
Factor 12 error	1556.80 (93.08)	*0.000*	4.242 (0.253)	*0.000*
Factor 13 error	1846.40 (110.10)	*0.000*	3.648 (0.218)	*0.000*
Factor 14 error	1989.10 (118.49)	*0.000*	3.742 (0.223)	*0.000*
Factor 15 error	1689.30 (100.87)	*0.000*	3.537 (0.211)	*0.000*
Factor 16 error	1887.00 (112.49)	*0.000*	3.645 (0.218)	*0.000*
Factor 17 error	1800.80 (107.42)	*0.000*	4.270 (0.254)	*0.000*
Factor 18 error	1859.50 (110.87)	*0.000*	3.941 (0.235)	*0.000*
Factor 19 error	1845.60 (110.05)	*0.000*	3.943 (0.235)	*0.000*
Factor 20 error	2010.60 (119.75)	*0.000*	3.447 (0.206)	*0.000*
**Reliability**	0.69		0.741	

Year 2007	OVER 65		UNDER 65	
**Fixed effects**	**Estimate (standard error)**	**P value**	**Estimate (standard error)**	**P value**
Intercept world	66.00 (2.17)	*0.00*	2.094 (0.063)	*0.000*
Region (rest)	18.60 (8.86)	*0.04*	0.046 (0.256)	*0.858*
Region Africa	19.40 (6.83)	*0.00*	2.135 (0.197)	*0.000*
Region east-med	7.24 (7.95)	*0.36*	0.638 (0.230)	*0.005*
Region Europe	-17.23 (6.73)	*0.01*	-0.384 (0.194)	*0.048*
Region America	29.91 (7.25)	*0.00*	0.436 (0.209)	*0.037*
Region SEAR	52.51 (9.55)	*0.00*	1.079 (0.276)	*0.000*
Factor 2	1.33 (3.13)	*0.67*	-0.114 (0.102)	*0.265*
Factor 3	-0.08 (3.13)	*0.98*	-0.010 (0.105)	*0.921*
Factor 4	-0.72 (3.10)	*0.82*	-0.008 (0.109)	*0.942*
Factor 5	0.09 (3.16)	*0.98*	0.018 (0.107)	*0.868*
Factor 6	1.11 (3.18)	*0.73*	-0.083 (0.102)	*0.416*
Factor 7	-4.21 (3.04)	*0.17*	-0.032 (0.107)	*0.763*
Factor 8	2.79 (3.20)	*0.38*	0.041 (0.104)	*0.691*
Factor 9	-1.15 (3.15)	*0.71*	-0.054 (0.105)	*0.607*
Factor 10	-2.56 (3.11)	*0.41*	0.081 (0.111)	*0.465*
Factor 11	-0.91 (3.16)	*0.77*	-0.120 (0.105)	*0.254*
Factor 12	0.83 (3.11)	*0.79*	0.050 (0.107)	*0.639*
Factor 13	-2.38 (3.11)	*0.44*	0.033 (0.108)	*0.757*
Factor 14	3.31 (3.22)	*0.30*	0.013 (0.107)	*0.905*
Factor 15	-2.09 (3.06)	*0.50*	-0.014 (0.105)	*0.895*
Factor 16	-1.65 (3.19)	*0.61*	-0.059 (0.103)	*0.566*
Factor 17	-0.01 (3.17)	*1.00*	-0.120 (0.105)	*0.252*
Factor 18	-2.49 (3.03)	*0.41*	-0.130 (0.099)	*0.191*
Factor 19	0.73 (3.16)	*0.82*	0.050 (0.105)	*0.638*
Factor 20	-3.51 (3.05)	*0.25*	0.000 (0.106)	*0.997*
**Random effects**	**Variance(standard**	**P value**	**Variance(standard**	**P value**

	error)		error)	
Between countries	599.37 (65.84)	0.000	0.486 (0.055)	*0.000*
Factor 1 error	2812.70 (167.89)	0.000	3.156 (0.188)	*0.000*
Factor 2 error	2872.90 (171.43)	0.000	2.865 (0.171)	*0.000*
Factor 3 error	2869.80 (171.25)	0.000	3.168 (0.189)	*0.000*
Factor 4 error	2752.10 (164.33)	0.000	3.774 (0.225)	*0.000*
Factor 5 error	2950.90 (176.02)	0.000	3.423 (0.204)	*0.000*
Factor 6 error	3042.80 (181.42)	0.000	2.922 (0.175)	*0.000*
Factor 7 error	2549.70 (152.43)	0.000	3.464 (0.206)	*0.000*
Factor 8 error	3128.40 (186.45)	0.000	3.160 (0.189)	*0.000*
Factor 9 error	2933.70 (175.01)	0.000	3.233 (0.193)	*0.000*
Factor 10 error	2801.90 (167.26)	0.000	3.979 (0.237)	*0.000*
Factor 11 error	2958.20 (176.44)	0.000	3.248 (0.194)	*0.000*
Factor 12 error	2785.10 (166.27)	0.000	3.496 (0.208)	*0.000*
Factor 13 error	2795.60 (166.89)	0.000	3.606 (0.215)	*0.000*
Factor 14 error	3196.90 (190.47)	0.000	3.418 (0.204)	*0.000*
Factor 15 error	2624.70 (156.84)	0.000	3.219 (0.192)	*0.000*
Factor 16 error	3071.30 (183.09)	0.000	2.958 (0.177)	*0.000*
Factor 17 error	2989.50 (178.28)	0.000	3.223 (0.192)	*0.000*
Factor 18 error	2494.80 (149.21)	0.000	2.559 (0.153)	*0.000*
Factor 19 error	2951.50 (176.05)	0.000	3.286 (0.196)	*0.000*
Factor 20 error	2574.10 (153.87)	0.000	3.392 (0.202)	*0.000*
**Reliability**	0.822		0.766	

**Year 2008**	**OVER 65**		**UNDER 65**	
**Fixed effects**	**Estimate (standard error)**	**P value**	**Estimate (standard error)**	**P value**
Intercept world	48.47 (1.41)	*0.00*	1.466 (0.057)	*0.000*
Region (rest)	15.72 (5.77)	*0.01*	0.222 (0.232)	*0.339*
Region Africa	16.66 (4.45)	*0.00*	1.242 (0.179)	*0.000*
Region east-med	17.55 (5.17)	*0.00*	0.673 (0.208)	*0.001*

Region Europe	0.93 (4.38)	*0.83*	-0.061 (0.176)	*0.730*
Region America	-1.52 (4.72)	*0.75*	0.149 (0.189)	*0.432*
Region SEAR	39.52 (6.22)	*0.00*	0.672 (0.249)	*0.007*
Factor 2	-1.00 (2.36)	*0.67*	-0.016 (0.083)	*0.847*
Factor 3	3.64 (2.52)	*0.15*	0.023 (0.086)	*0.794*
Factor 4	3.72 (2.49)	*0.14*	0.115 (0.087)	*0.183*
Factor 5	2.83 (2.50)	*0.26*	0.015 (0.081)	*0.853*
Factor 6	1.90 (2.47)	*0.44*	0.069 (0.085)	*0.417*
Factor 7	4.89 (2.44)	*0.04*	0.062 (0.085)	*0.466*
Factor 8	0.81 (2.38)	*0.73*	-0.051 (0.084)	*0.547*
Factor 9	5.37 (2.63)	*0.04*	0.068 (0.083)	*0.411*
Factor 10	1.11 (2.43)	*0.65*	-0.006 (0.086)	*0.948*
Factor 11	2.13 (2.39)	*0.37*	-0.041 (0.079)	*0.603*
Factor 12	3.14 (2.53)	*0.21*	-0.045 (0.082)	*0.578*
Factor 13	-0.49 (2.41)	*0.84*	-0.057 (0.081)	*0.480*
Factor 14	3.10 (2.53)	*0.22*	0.044 (0.082)	*0.597*
Factor 15	1.77 (2.43)	*0.47*	-0.027 (0.083)	*0.746*
Factor 16	4.37 (2.59)	*0.09*	0.082 (0.088)	*0.352*
Factor 17	4.07 (2.58)	*0.12*	0.006 (0.082)	*0.946*
Factor 18	-0.50 (2.39)	*0.83*	0.133 (0.086)	*0.123*
Factor 19	4.39 (2.60)	*0.09*	0.063 (0.086)	*0.464*
Factor 20	3.68 (2.50)	*0.14*	-0.032 (0.082)	*0.695*
**Random effects**	**Variance(standard error)**	**P value**	**Variance(standard error)**	**P value**
Between countries	242.35 (27.92)	*0.000*	0.408 (0.045)	*0.000*
Factor 1 error	1615.00 (96.57)	*0.000*	2.043 (0.122)	*0.000*
Factor 2 error	1608.40 (96.18)	*0.000*	1.983 (0.118)	*0.000*
Factor 3 error	2061.80 (122.83)	*0.000*	2.266 (0.135)	*0.000*
Factor 4 error	1983.10 (118.21)	*0.000*	2.299 (0.137)	*0.000*
Factor 5 error	2011.50 (119.87)	*0.000*	1.803 (0.108)	*0.000*

Factor 6 error	1905.90 (113.67)	*0.000*	2.168 (0.129)	*0.000*
Factor 7 error	1819.30 (108.58)	*0.000*	2.111 (0.126)	*0.000*
Factor 8 error	1663.00 (99.40)	*0.000*	2.031 (0.121)	*0.000*
Factor 9 error	2397.30 (142.55)	*0.000*	1.921 (0.115)	*0.000*
Factor 10 error	1796.10 (107.21)	*0.000*	2.192 (0.131)	*0.000*
Factor 11 error	1682.00 (100.51)	*0.000*	1.566 (0.094)	*0.000*
Factor 12 error	2085.00 (124.20)	*0.000*	1.805 (0.108)	*0.000*
Factor 13 error	1747.10 (104.33)	*0.000*	1.786 (0.107)	*0.000*
Factor 14 error	2077.20 (123.75)	*0.000*	1.896 (0.113)	*0.000*
Factor 15 error	1807.30 (107.88)	*0.000*	1.913 (0.114)	*0.000*
Factor 16 error	2265.40 (134.80)	*0.000*	2.401 (0.143)	*0.000*
Factor 17 error	2251.50 (133.99)	*0.000*	1.871 (0.112)	*0.000*
Factor 18 error	1682.70 (100.55)	*0.000*	2.235 (0.133)	*0.000*
Factor 19 error	2295.40 (136.56)	*0.000*	2.252 (0.134)	*0.000*
Factor 20 error	2017.70 (120.24)	*0.000*	1.808 (0.108)	*0.000*
**Reliability**	0.734		0.82	

**Year 2010**	**OVER 65**		**UNDER 65**	
**Fixed effects**	**Estimate (standard error)**	**P value**	**Estimate (standard error)**	**P value**
Intercept world	35.63 (1.33)	*0.00*	1.881 (0.047)	*0.000*
Region (rest)	11.89 (5.45)	*0.03*	-0.221 (0.190)	*0.245*
Region Africa	5.22 (4.20)	*0.21*	1.034 (0.147)	*0.000*
Region east-med	14.68 (4.88)	*0.00*	0.271 (0.170)	*0.112*
Region Europe	-10.49 (4.14)	*0.01*	-0.435 (0.144)	*0.003*
Region America	-8.92 (4.46)	*0.05*	-0.593 (0.156)	*0.000*
Region SEAR	35.70 (5.87)	*0.00*	1.026 (0.205)	*0.000*
Factor 2	1.48 (1.90)	*0.44*	0.020 (0.117)	*0.862*
Factor 3	0.28 (1.89)	*0.88*	-0.062 (0.110)	*0.574*
Factor 4	0.76 (1.91)	*0.69*	-0.049 (0.111)	*0.657*
Factor 5	-1.68 (1.77)	*0.34*	-0.108 (0.109)	*0.321*

Factor 6	0.71 (1.85)	*0.70*	0.077 (0.122)	*0.529*
Factor 7	2.38 (1.85)	*0.20*	-0.023 (0.113)	*0.837*
Factor 8	-1.25 (1.80)	*0.49*	-0.059 (0.112)	*0.601*
Factor 9	1.47 (1.84)	*0.42*	-0.101 (0.109)	*0.355*
Factor 10	2.04 (1.89)	*0.28*	-0.024 (0.114)	*0.832*
Factor 11	1.68 (1.92)	*0.38*	-0.068 (0.110)	*0.536*
Factor 12	1.56 (1.85)	*0.40*	-0.052 (0.111)	*0.643*
Factor 13	-0.82 (1.76)	*0.64*	-0.055 (0.110)	*0.618*
Factor 14	-0.99 (1.82)	*0.59*	-0.132 (0.107)	*0.218*
Factor 15	0.87 (1.89)	*0.65*	0.045 (0.116)	*0.700*
Factor 16	-0.30 (1.77)	*0.87*	-0.035 (0.114)	*0.760*
Factor 17	1.93 (1.93)	*0.32*	-0.053 (0.114)	*0.640*
Factor 18	1.00 (1.88)	*0.59*	0.055 (0.113)	*0.624*
Factor 19	-1.60 (1.71)	*0.35*	-0.010 (0.111)	*0.925*
Factor 20	1.27 (1.90)	*0.50*	-0.040 (0.114)	*0.723*
**Random effects**	**Variance(standard error)**	**P value**	**Variance(standard error)**	**P value**
Between countries	227.20 (24.88)	*0.000*	0.241 (0.030)	*0.000*
Factor 1 error	924.72 (55.29)	*0.000*	3.929 (0.234)	*0.000*
Factor 2 error	1173.70 (69.92)	*0.000*	3.998 (0.238)	*0.000*
Factor 3 error	1139.10 (67.89)	*0.000*	3.134 (0.187)	*0.000*
Factor 4 error	1186.30 (70.66)	*0.000*	3.257 (0.194)	*0.000*
Factor 5 error	879.37 (52.62)	*0.000*	2.959 (0.177)	*0.000*
Factor 6 error	1056.30 (63.02)	*0.000*	4.698 (0.279)	*0.000*
Factor 7 error	1065.40 (63.56)	*0.000*	3.400 (0.202)	*0.000*
Factor 8 error	953.31 (56.97)	*0.000*	3.336 (0.199)	*0.000*
Factor 9 error	1037.70 (61.93)	*0.000*	3.013 (0.180)	*0.000*
Factor 10 error	1136.20 (67.72)	*0.000*	3.557 (0.212)	*0.000*
Factor 11 error	1201.10 (71.53)	*0.000*	3.029 (0.181)	*0.000*
Factor 12 error	1047.50 (62.50)	*0.000*	3.220 (0.192)	*0.000*

Factor 13 error	875.22 (52.38)	*0.000*	3.046 (0.182)	*0.000*
Factor 14 error	996.86 (59.53)	*0.000*	2.745 (0.164)	*0.000*
Factor 15 error	1150.20 (68.54)	*0.000*	3.871 (0.230)	*0.000*
Factor 16 error	890.73 (53.29)	*0.000*	3.604 (0.214)	*0.000*
Factor 17 error	1220.90 (72.69)	*0.000*	3.643 (0.217)	*0.000*
Factor 18 error	1120.00 (66.77)	*0.000*	3.444 (0.205)	*0.000*
Factor 19 error	773.52 (46.40)	*0.000*	3.155 (0.188)	*0.000*
Factor 20 error	1160.40 (69.14)	*0.000*	3.533 (0.210)	*0.000*
**Reliability**	0.827		0.61	

**Year 2011**	**OVER 65**		**UNDER 65**	
**Fixed effects**	**Estimate (standard error)**	**P value**	**Estimate (standard error)**	**P value**
Intercept world	48.51 (1.07)	*0.00*	1.592 (0.050)	*0.000*
Region (rest)	-4.06 (4.38)	*0.35*	-0.023 (0.205)	*0.910*
Region Africa	-2.10 (3.38)	*0.53*	1.407 (0.158)	*0.000*
Region east-med	-7.55 (3.93)	*0.05*	0.460 (0.184)	*0.012*
Region Europe	-17.67 (3.33)	*0.00*	-0.267 (0.156)	*0.086*
Region America	-24.43 (3.58)	*0.00*	-0.448 (0.168)	*0.008*
Region SEAR	35.79 (4.72)	*0.00*	0.972 (0.221)	*0.000*
Factor 2	-1.39 (2.31)	*0.55*	0.035 (0.081)	*0.662*
Factor 3	-0.81 (2.36)	*0.73*	-0.009 (0.079)	*0.906*
Factor 4	0.91 (2.40)	*0.71*	-0.028 (0.079)	*0.719*
Factor 5	-0.19 (2.33)	*0.94*	0.078 (0.081)	*0.336*
Factor 6	0.92 (2.36)	*0.70*	0.071 (0.081)	*0.385*
Factor 7	-3.20 (2.27)	*0.16*	0.054 (0.081)	*0.504*
Factor 8	-0.74 (2.31)	*0.75*	0.034 (0.081)	*0.680*
Factor 9	-0.26 (2.32)	*0.91*	-0.021 (0.083)	*0.801*
Factor 10	-0.08 (2.37)	*0.97*	0.041 (0.083)	*0.618*
Factor 11	-3.15 (2.30)	*0.17*	0.107 (0.082)	*0.191*
Factor 12	-0.11 (2.37)	*0.96*	0.010 (0.079)	*0.898*

Factor 13	-0.44 (2.33)	*0.85*	0.023 (0.082)	*0.776*
Factor 14	0.03 (2.39)	*0.99*	0.006 (0.077)	*0.938*
Factor 15	-1.86 (2.29)	*0.42*	0.002 (0.080)	*0.982*
Factor 16	-0.09 (2.36)	*0.97*	-0.006 (0.084)	*0.940*
Factor 17	-0.12 (2.38)	*0.96*	0.093 (0.081)	*0.251*
Factor 18	-0.48 (2.39)	*0.84*	0.064 (0.083)	*0.438*
Factor 19	0.53 (2.37)	*0.82*	0.134 (0.084)	*0.113*
Factor 20	-2.12 (2.28)	*0.35*	-0.020 (0.079)	*0.801*
**Random effects**	**Variance(standard error)**	**P value**	**Variance(standard error)**	**P value**
Between countries	131.26 (16.09)	*0.000*	0.314 (0.035)	*0.000*
Factor 1 error	1559.90 (92.99)	*0.000*	1.849 (0.110)	*0.000*
Factor 2 error	1527.70 (91.10)	*0.000*	1.959 (0.117)	*0.000*
Factor 3 error	1677.50 (99.90)	*0.000*	1.722 (0.103)	*0.000*
Factor 4 error	1785.40 (106.24)	*0.000*	1.732 (0.104)	*0.000*
Factor 5 error	1596.30 (95.13)	*0.000*	1.942 (0.116)	*0.000*
Factor 6 error	1678.10 (99.93)	*0.000*	1.985 (0.118)	*0.000*
Factor 7 error	1436.00 (85.71)	*0.000*	1.923 (0.115)	*0.000*
Factor 8 error	1542.10 (91.94)	*0.000*	1.986 (0.118)	*0.000*
Factor 9 error	1556.70 (92.80)	*0.000*	2.128 (0.127)	*0.000*
Factor 10 error	1686.10 (100.40)	*0.000*	2.123 (0.127)	*0.000*
Factor 11 error	1499.70 (89.45)	*0.000*	2.029 (0.121)	*0.000*
Factor 12 error	1683.50 (100.25)	*0.000*	1.747 (0.104)	*0.000*
Factor 13 error	1593.90 (94.99)	*0.000*	2.016 (0.120)	*0.000*
Factor 14 error	1736.40 (103.36)	*0.000*	1.618 (0.097)	*0.000*
Factor 15 error	1469.60 (87.68)	*0.000*	1.882 (0.112)	*0.000*
Factor 16 error	1664.30 (99.13)	*0.000*	2.244 (0.134)	*0.000*
Factor 17 error	1730.10 (102.99)	*0.000*	1.918 (0.114)	*0.000*
Factor 18 error	1741.30 (103.65)	*0.000*	2.106 (0.125)	*0.000*
Factor 19 error	1694.50 (100.90)	*0.000*	2.263 (0.135)	*0.000*

Factor 20 error	1437.20 (85.78)	*0.000*	1.763 (0.105)	*0.000*
**Reliability**	0.642		0.781	

***** Note (applying to all model outputs in Table 3): The 20 Factors represent the 20 imputed datasets (as the Stage 2 procedure produces 20 imputed data points per country per year, and each dataset has a different average) which are included in the hierarchical linear random effects regression model to calculate the point estimates (with standard error) by country, region and the world, see the formula of the data analysis step below, where f stands for imputed dataset. "Factor n" in the table above represents the term $\beta_f X$ and "Factor n error" represents ε_f .

The Stage 2 model contains many components, many of which are nuisance parameters used to control for bias and to adjust for the way the datasets are created. Useful fixed effects components are the 'Intercept world,' which yields the global rate, and the 'Regional' parameters (e.g. Africa and Eastern Mediterranean), which yield the regional rates, and the 'Between countries' variance, which yields the random effects component. Note that overall fit statistics (e.g. R^2) have little meaning here due to the large number of nuisance parameters; we used the reliability coefficient instead.

Table S4: Predictors of influenza-related excess mortality rates by age group and country.

Same as in Main text Table 3 but for the subset of countries that have Stage 1 estimates. Results of a multivariate mixed generalized linear regression model applied to 28 countries for which vital statistics were modeled to assess influenza burden over 9 years, 2002-2011, after exclusion of the pandemic period. Best model selected by Akaike's information criterion (AIC). This analysis does not take into account the errors in Stage 1 estimates

	**Under 65 years.** Estimate (SE) Significance*	**65 years & above** Estimate (SE) Significance*
Intercept	7.09 (1.04) †††	-33.5 (43.04)
Health and socio-economic development:		
HAQI †	-0.03 (0.02)	
Baseline respiratory death rate ‡		3.56 (1.24) †
Viral characteristics:		
Mixed season	1.00 (ref)	1.00 (ref)
Dominant A/H1N1	0.14 (0.15)	-1.4 (6.46)
Dominant A/H1N1pdm	0.53 (0.14) ††	-4.49 (6.25)
Dominant A/H3N2	0.2 (0.08) †	12.71 (3.5) ††
Region:		
Sub-Saharan Africa	1.00 (ref)	1.00 (ref)
Eastern Mediterranean	N/A	N/A
Europe	-4.44 (0.85) †††	15.11 (34.28)
Americas	-3.89 (0.74) †††	32.34 (31.82)
South-East Asia	-4.35 (0.94) †††	9.47 (40.03)
Western Pacific	-4.22 (0.88) †††	17.88 (33.14)

* Significance level: †, *<0.05*; ††, *<0.001*; †††, *<0.0001*. We used the Kenward-Roger approximation to obtain approximate degrees of freedom for the mixed model, and the *t*-distribution for *p*-values.

† Healthcare And Quality Index (HAQI), reflecting amenable mortality causes (32 causes were considered). Higher values indicate higher healthcare access and quality.

‡ Source: Institute for Health Metrics and Evaluation

Figure S1: Comparison of country-specific annual influenza-associated death rates for 31 countries with Stage 1 data and the imputed estimates resulting from Stage 2 approach.

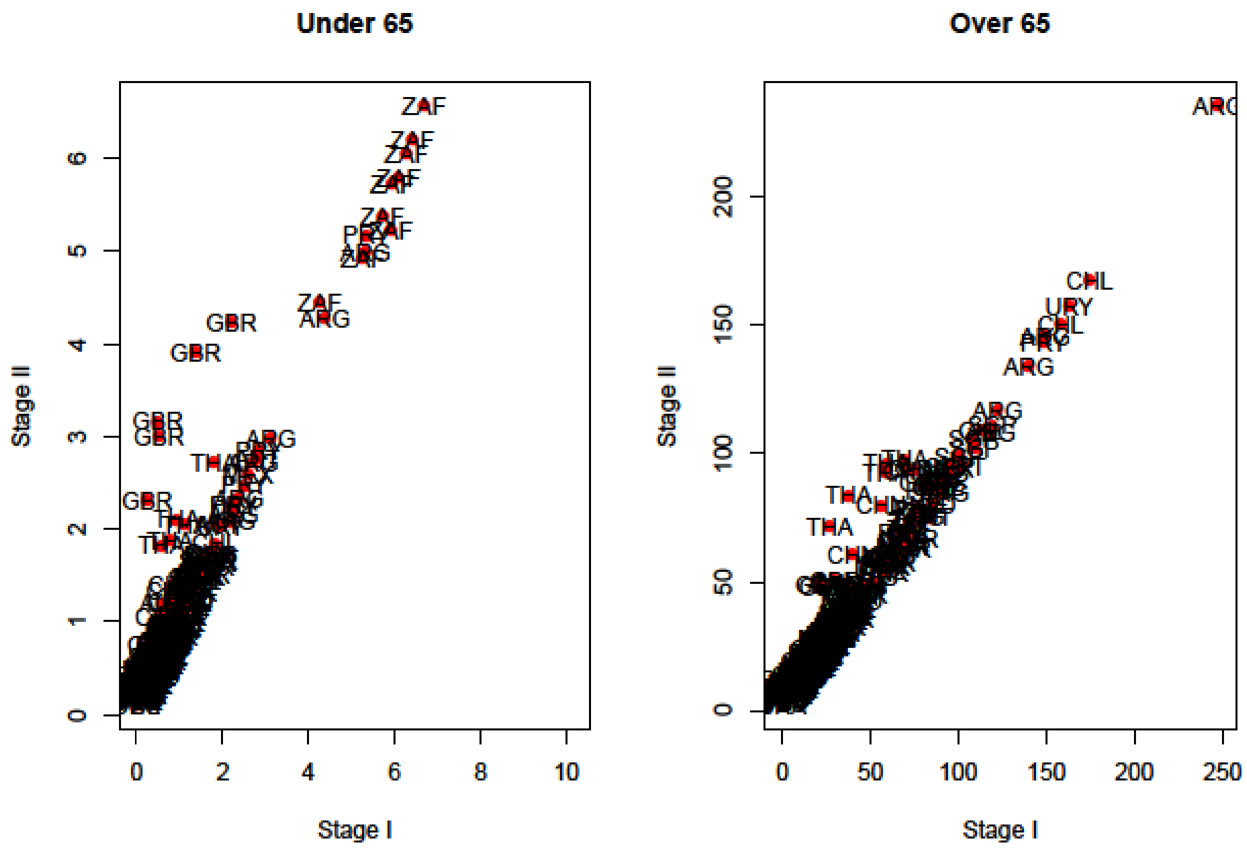

Figure S2: Relationship between influenza-associated -related death rates and total respiratory death rates (annual country-specific Stage 2 estimates).

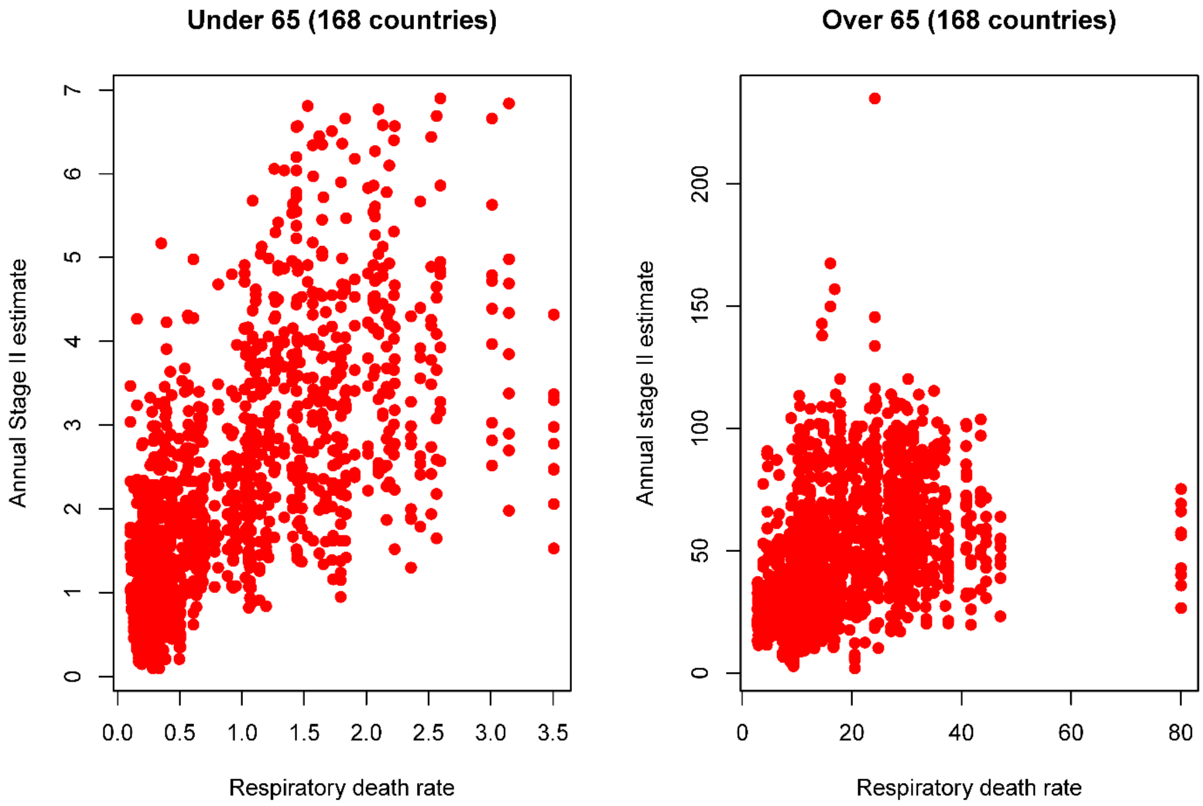

Figure S3: Relationship between influenza-associated death rates and total respiratory death rates (Stage 1 sample).

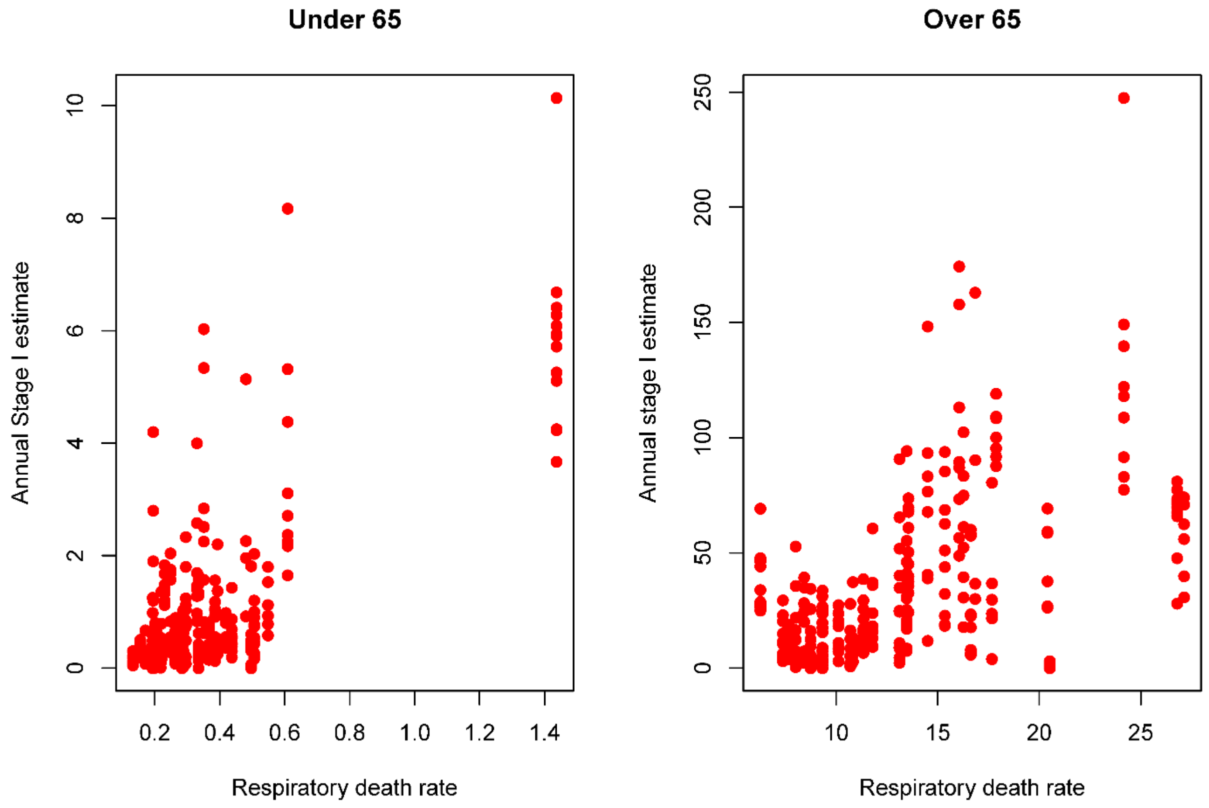

Figure S4: Proportion of total respiratory deaths attributed to influenza (Stage 1 sample).

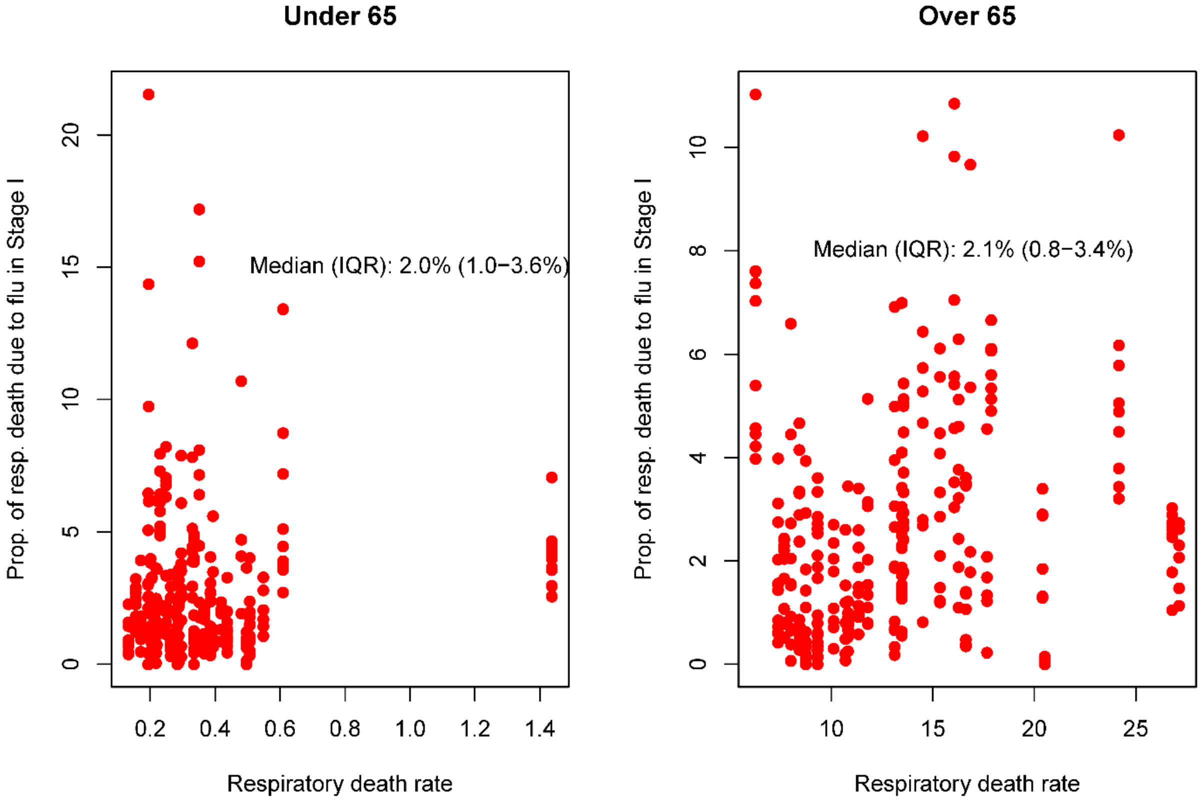

Supplement: Online Supplementary Document [file jogh-09-020421-s001.pdf]
